# Enteral nutrition interruption in critically ill patients: a systematic review and meta-analysis of prevalence, causes, and clinical outcomes

**DOI:** 10.3389/fnut.2026.1886962

**Published:** 2026-09-24

**Authors:** Xiaojing Guo, Libing Jiang, Shouyin Jiang, Xiaoxia Huang, Jiaying Tang, Xiaoli Liang, Xiuqin Feng, Shanxiang Xu

**Affiliations:** 1 Department of Nursing, The Second Affiliated Hospital of Zhejiang University School of Medicine, Hangzhou, China; 2 Department of Nursing, Zhejiang University School of Medicine, Hangzhou, China; 3 Department of Emergency Medicine, Second Affiliated Hospital, Zhejiang University School of Medicine, Hangzhou, China; 4 Zhejiang Key Laboratory of Trauma, Burn, and Medical Rescue, Hangzhou, China; 5 Zhejiang Province Clinical Research Center for Emergency and Critical Care Medicine, Hangzhou, China; 6 Research Institute of Emergency Medicine, Zhejiang University, Hangzhou, China; 7 National Emergency Medical Rescue Base, Hangzhou, China

**Keywords:** critically ill, enteral nutrition, enteral nutrition interruption, intensive care unit, meta-analysis

## Abstract

**Introduction:**

Enteral nutrition interruption (ENI) is common in critically ill patients and may compromise nutritional delivery, but its prevalence, causes, and clinical consequences remain uncertain. This systematic review and meta-analysis synthesized evidence on ENI prevalence, contributing factors, clinical outcomes, and potential interventions.

**Methods:**

PubMed, Embase, CINAHL, Web of Science, Cochrane Library, CNKI, and WanFang Data were searched from inception through December 2025. Studies of critically ill adults receiving enteral nutrition were eligible. The review followed PRISMA guidance and was registered in PROSPERO (CRD420251266654). Random-effects models, subgroup analyses, sensitivity analyses, and GRADE assessments were performed.

**Results:**

Forty-one studies involving 8,260 critically ill patients were included. Crude ENI prevalence ranged from 11.2% to 100%, with a median of 68.5% (IQR, 57.1%-96.6%). The estimated pooled prevalence was 79.2%, although heterogeneity was extreme (*I*^2^ = 99%) and publication bias was evident (Egger’s test, p < 0.001). Common causes included airway management, radiological or imaging procedures, gastrointestinal intolerance, and surgical procedures or preparation. ENI was associated with reduced energy adequacy (mean difference, -4.21 percentage points; 95% CI, –7.71 to –0.71; *p* = 0.02). No statistically significant association was observed with ICU length of stay (mean difference, –0.41 days; 95% CI, –2.05 to 1.23; *p* = 0.62). Short-term mortality was not statistically significantly associated with ENI (risk ratio, 1.86; 95% CI, 0.67–5.11), but the estimate was imprecise and heterogeneous (*I*^2^ = 88%).

**Discussion:**

ENI appears common and is predominantly procedure-related. It is associated with reduced energy adequacy, whereas its effects on ICU length of stay and short-term mortality remain uncertain because of inconsistent ENI definitions, substantial heterogeneity, publication bias, and limited outcome data. Standardized definitions, shorter peri-procedural fasting, improved gastrointestinal management, and multidisciplinary nutrition protocols may reduce avoidable interruptions. High-quality prospective studies and randomized trials are needed.

**Systematic review registration:**

https://www.crd.york.ac.uk/PROSPERO/view/CRD420251266654.

## Introduction

Adequate nutritional support is a cornerstone of management in critically ill patients (1), with early enteral nutrition (EN) recommended by international guidelines to preserve gut integrity, modulate immune responses, and improve clinical outcomes (2, 3). However, despite strong guideline endorsement, the delivery of EN in the intensive care unit (ICU) remains suboptimal, 60–75% of patients in the ICU (4, 5), do not reach the target feeding volume. Research has found that the feeding deficiency rates were 54 and 15% (*p* < 0.001) on trial days with and without ENI (6), respectively, indicating a positive correlation between ENI and insufficient feeding. The incidence of ENI is relatively high in the ICU, ranging from 54 to 84% (1).

Although ENI has been increasingly recognized as an important clinical issue, existing studies are highly heterogeneous in terms of definitions, reported prevalence, contributing factors, and outcome measures. Reported ENI rates vary widely across studies, partly due to inconsistent definitions of interruption duration and differences in clinical practice. Moreover, while several studies have explored the causes of ENI, there is no comprehensive synthesis of these factors across different ICU settings. In addition, evidence regarding clinical and biochemical indicators associated with ENI remains limited and inconsistently reported, and the effectiveness of proposed interventions has not been systematically evaluated.

Therefore, a comprehensive synthesis of the current evidence is needed to better understand the burden, determinants, and potential clinical consequences of ENI in critically ill patients. This systematic review and meta-analysis aims to (1) estimate the pooled prevalence of ENI, (2) identify the main contributing factors, (3) summarize reported clinical outcomes (including energy adequacy, intensive care unit length of stay (LOS), and short-term mortality) and biochemical indicators, and (4) synthesize evidence on potential intervention strategies to reduce interruptions and optimize nutritional delivery in the ICU.

## Methods

This study was performed according to the Preferred Reporting Items for Systematic Reviews and Meta-Analyses (PRISMA) checklist (7). This systematic review was registered prospectively with the PROSPERO database (registration number: CRD420251266654) on 16 December 2025.

### Search strategy

We performed a systematic search of the PubMed, Embase, CINAHL, Web of Science, Cochrane Library, CNKI, and WanFang Data electronic databases. References were also traced. Only original studies published in English or Chinese were included, and the search spanned from database inception to December, 2025. The search employed a combination of Medical Subject Headings (MeSH) and free-text keywords related to: “Intensive Care Units,” “critically ill patients,” “ICU,” “Enteral Nutrition,” “tube feeding,” “nutrition support,” “Treatment Interruption,” “feeding interruption,” “enteral nutrition interruption.” The whole search strategy is provided in Supplementary Tables S1–S7.

### Inclusion and exclusion criteria

This review included studies that met the following PICOS criteria:

Population (P): Critically ill adult patients (aged ≥18 years) admitted to intensive care units receiving enteral nutrition support. Intervention (I): Clinical situations featuring ENI, including all types, durations and root causes of feeding suspension. Comparator (C): Critically ill adult patients receiving continuous enteral nutrition without any recorded ENI episodes. However, considering that this article is a meta-analysis of ENI incidence rates, the absence of a control group is also acceptable. Outcome (O): Primary outcome: Pooled prevalence of ENI and energy adequacy rate; Secondary outcomes: ICU length of stay, short-term all-cause mortality, serum nutritional and inflammatory biochemical markers. Study design (S): Observational studies (cohort, cross-sectional), randomized controlled trials and their secondary analyses published in Chinese or English.

Study exclusion criteria included inaccessible full text, duplicate publications or studies based on the same patient cohort, incomplete or unextractable study data, reviews, editorials, conference abstracts, case reports, and studies with low-quality results assessed by critical appraisal tools.

### Study selection and data extraction

Based on the search results, the literature was first imported into the reference manager EndNote X9 for deduplication. Two researchers then independently screened the literature according to the inclusion and exclusion criteria. The screening process involved initially reviewing the titles and abstracts, followed by reading the full texts to determine eligibility. Additionally, the references of included articles were manually searched to identify further relevant studies. In cases where consensus could not be reached, a third researcher was consulted. Extracted data from included studies comprised study identifier, country, department, sample size, number of centers, age, study design, incidence of ENI, definition of ENI, feeding route, biochemical markers, causes of ENI, and recommended interventions.

### Risk of bias assessment

Two reviewers independently performed risk of bias assessments for the included studies. All reviews underwent cross-verification procedures, with disagreements resolved through a third reviewer. Cohort and case–control studies were assessed using the Newcastle–Ottawa Scale (NOS), which has three dimensions: participant selection, group comparability, and outcome assessment, with a total score of 9. Study quality scores were defined arbitrarily as poor (0–3), fair (4–6), or good (7–9) (8). Cross-sectional studies used the 11-item AHRQ checklist (1 = compliant, 0 = unclear/non-compliant), categorized as high (8–11), moderate (4–7), or low quality (0–3). RCTs and their secondary analyses were evaluated with Cochrane ROB 2.0, rating seven bias domains to determine “low risk,” “some concerns,” or “high risk.” Two reviewers independently assessed quality, resolving disagreements via discussion or third-party arbitration (9).

### Certainty of evidence assessment

The certainty of evidence related to our primary outcomes was evaluated using the Grading of Recommendations Assessment, Development, and Evaluation (GRADE) (10) approach to categorize the certainty of evidence as “high,” “moderate,” “low,” or “very low.” As part of the GRADE assessment, evidence was evaluated based on risk of bias, inconsistency, indirectness, imprecision, and other biases including publication bias and small study effects (by examining funnel plot asymmetry). Any discrepancies between author assessments were resolved by consensus.

### Data analysis

Statistical analyses were performed using Review Manager (RevMan) version 5.4 (The Cochrane Collaboration, Copenhagen, Denmark) and Stata version 17.0 (StataCorp LLC, College Station, TX, USA). A single-group rate meta-analysis with logit transformation was conducted to estimate the pooled prevalence of ENI across all included studies. Heterogeneity across studies was evaluated using the *I*^2^ statistic, with *I*^2^ > 50% indicating substantial heterogeneity, and a random-effects model was used for all analyses. Subgroup analyses were performed to explore sources of heterogeneity, stratified by study design, ICU type, country development level, ENI time threshold definition, feeding route, and feeding mode. Sensitivity analysis was carried out by sequentially omitting one study at a time to assess the stability of the pooled effect estimates. Publication bias was visually assessed using funnel plots and quantitatively assessed using Egger’s linear regression test, with a two-sided *p* < 0.05 considered statistically significant.

## Results

### Selection of trials

The systematic search initially retrieved 5,113 citations from 7 databases and registers. After removing 826 duplicates, 4,218 irrelevant studies were excluded via title/abstract screening, leaving 69 records for abstract assessment. Of these, 31 proceeded to full-text review, where 38 were excluded (8 for topic mismatch, 15 for inconsistent subjects, 10 for inappropriate study type, 2 for unavailable full text, 3 for inconsistent data types). An additional 10 eligible studies were identified from other sources, resulting in 41 included studies. The selection process is detailed in Figure 1.

**Figure 1:**
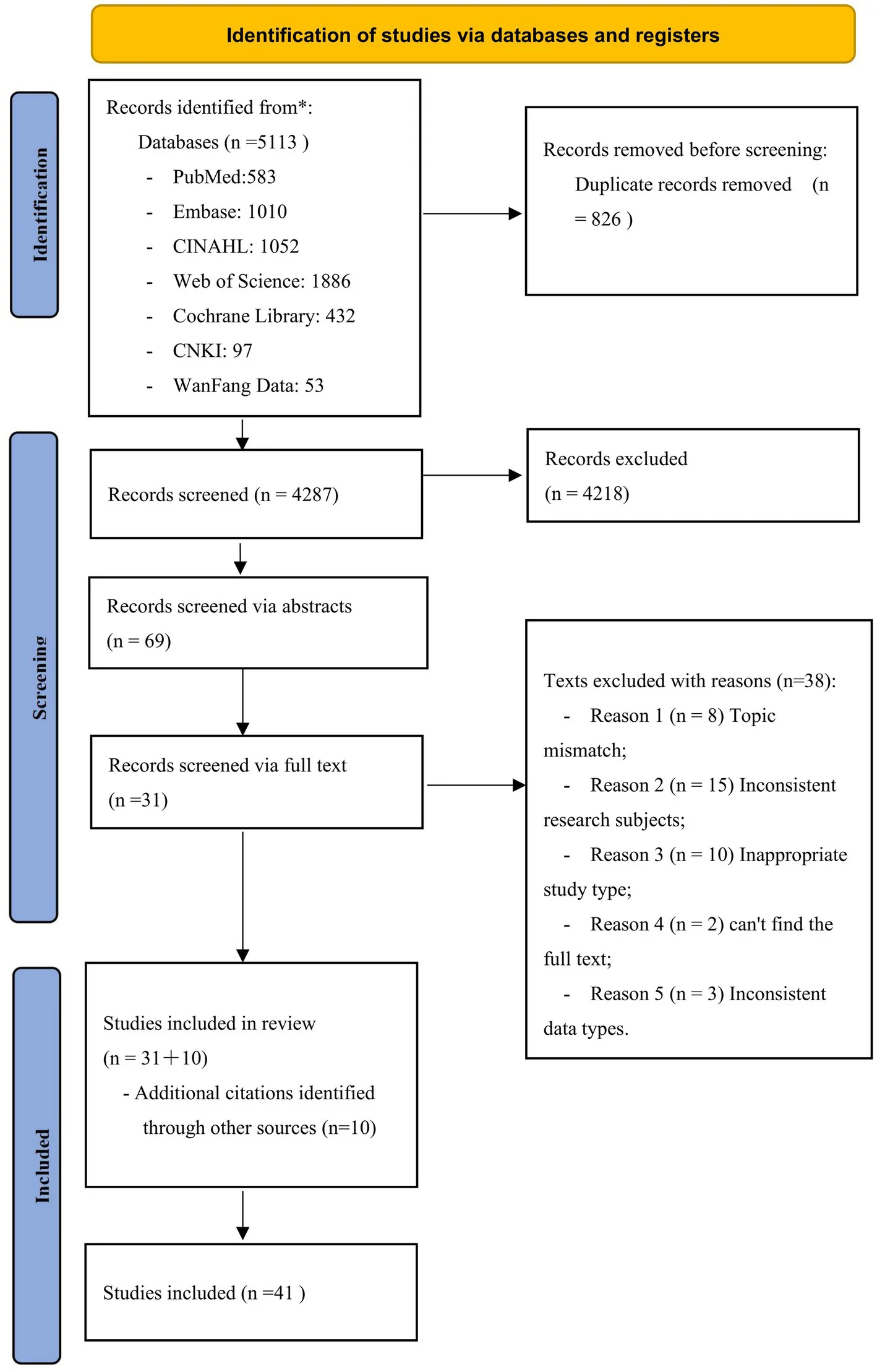
PRISMA flow diagram of study selection.

### Trial characteristics

The basic characteristics of the included studies are presented in Tables 1a,b. A total of 41 studies (6, 11–50) involving 8,260 critically ill patients receiving EN were included, with sample sizes ranging from 22 to 1,495 participants (35, 44). The included patients had a median age between 51 and 64 years across studies, with some studies reporting that most patients were under 60 years old. Only a few studies were multicenter investigations, including Davies et al. (25) involving 40 centers, Zheng et al. (21) involving 90 centers, and the large international observational study by Saran et al. (35) involving 403 centers, as well as the international multicenter study reported by Czapran et al. (36).

**Table 1a:** Baseline characteristics of included studies.

Study	Country	Department	Sample size (*n*)	Number of centers	Age (Mean ± SD)	Study design
Kasti et al. (32)	Greece	ICU	81	1	64 ± 15	Prospective observational study
Arunkumar et al. (41)	India	ICU	622	1	55.8 ± 17.8	Prospective observational study
Binnekade et al. (24)	The Netherlands	ICU	403	1	60 ± 17	Retrospective database study
Davies et al. (25)	UK	ICU	732	4	53.2 ± 20.4	Prospective observational study (secondary analysis)
Ritter et al. (49)	Brazil	ICU	130	1	73.8% were < 60 years, 26.2% were ≥ 60 yearsMean age not reported	Prospective cohort study
Coutris et al. (37)	Canada	Neurocritical ICU	27	1	Median: 51 (IQR:36–61) years	Retrospective observational study
Czapran et al. (36)	International	ICU	90	Multi-center	41 ± 17	Prospective observational study
Davies et al. (43)	Australia	ICU	58	40	61.3 ± 17.2	Prospective observational study
Segaran et al. (44)	UK	ICU	22	1	58.5 ± 29.5	Prospective observational study
Elpern et al. (45)	USA	ICU	39	1	60.6 years (range 27–93 years)	Prospective descriptive study
Erika et al. (6)	Lithuania	ICU	73	1	64 ± 13	Prospective observational study
Fang (15)	China	Neurological ICU	30	1	59.59 ± 14.26	Cross-sectional study
Gong (14)	China	ICU	198	1	51.56 ± 15.10	Cross-sectional study
Habib et al. (39)	Pakistan	Medical ICU	106	1	61.83 ± 11.21	Prospective observational study
Han et al. (11)	China	Surgical ICU	91	1	72 cases (79.12%) ≤ 60 years old and 19 cases (20.88%) > 60 years old	Retrospective cohort study
Zheng et al. (21)	China	ICU	1,331	90	Median age 64 years [interquartile range (IQR): 49–76 years]	Randomized controlled trial(secondary analysis)
Kim et al. (19)	Korea	Neurological ICU	47	1	56.62 ± 16.37	Prospective descriptive study
Pinilla et al. (22)	Canada	ICU	80	1	Group I: 49.1 ± 21.6 years; Group II: 56.7 ± 16.9 years	Randomized controlled trial
Kim et al. (28)	South Korea	Medical ICU	34	1	70.8 ± 14.8	Prospective cohort study
Lakshmi and Vaidyanathan (40)	India	Medical ICU	25	1	49 ± 8	Prospective observational study
O’Leary-Kelley et al. (42)	USA	ICU	60	2	63.9 ± 12.9	Prospective descriptive study
Li (17)	Brazil	ICU	166	1	57.94 ± 1.27	Cross-sectional study
Ribeiro et al. (18)	China	ICU	93	1	55.7 ± 17.4	Prospective observational study
McClave et al. (29)	USA	ICU	44	2	Mean age: 57.8	Prospective observational study
O’Meara et al. (30)	USA	Medical ICU	59	1	56.5 ± 13.9	Prospective observational study
Uozumi et al. (38)	Japan	ICU	100	1	66.7 ± 14.4	Retrospective observational study
Peev et al. (20)	USA	Surgical ICU	94	1	63 ± 17	Prospective observational study
Van Nieuwkoop et al. (27)	The Netherlands	ICU	61	1	60.8 ± 14.3	Prospective observational study
Morgan et al. (33)	USA	Surgical ICU	56	1	40 ± 16	Retrospective observational study
Daphnee et al. (23)	India	ICU	327	1	57.95 ± 16.95	Prospective observational study
Passier et al. (46)	Australia	ICU	41	1	45.0 ± 19.3	Retrospective observational study
Saran et al. (35)	International	ICU	1,495	403	53.0 ± 19.0	Prospective observational study
Onuk et al. (26)	Turkey	Medical ICU	122	1	Median age 63 years [IQR: 49–75 years]	Prospective observational study
Shankar et al. (34)	India	ICU	308	1	55.59 ± 17.87	Prospective observational study
Williams et al. (48)	Australia	ICU	505	1	Median age 53 years (IQR 35–65 years)	Prospective comparative study (before–after)
Rice et al. (50)	USA	ICU	55	1	Mean age: 50.6 years	Prospective observational study
Wang and Li (16)	China	Neurocritical ICU	84	1	64.89 ± 7.51	Prospective observational study
Yip et al. (31)	Malaysia	ICU	77	1	51.0 ± 16.6	Cross-sectional study
Zheng et al. (13)	China	ICU	31	1	72.4 ± 7.82	Prospective cohort study
Lee et al. (47)	Malaysia	ICU	148	1	51.3 ± 15.6	Prospective observational study (secondary analysis)
Zuo et al. (12)	China	Surgical ICU	115	1	45.90 ± 14.37	Retrospective observational study

### Quality evaluation of the literature

Among the 41 included studies, 35 observational studies were assessed using the NOS, with scores ranging from 7 to 8, indicating generally high methodological quality (Supplementary Table S8). Four cross-sectional studies (14, 15, 17, 31) were evaluated using the AHRQ checklist, with scores ranging from 9 to 10, suggesting moderate-to-high quality (Supplementary Table S9). Two RCTs (21, 22) or secondary analyses of randomized trials were assessed using the Cochrane ROB 2.0 tool and were judged to have some concerns of bias mainly due to the lack of participant blinding (Supplementary Table S10).

### Prevalence of ENI

The unpooled crude incidence of ENI demonstrated tremendous between-study fluctuation, ranging from 11.2 to 100%. The median ENI prevalence across individual investigations was 68.5%, with an interquartile range of 57.1–96.6%. These findings indicate that the pooled global estimate should not be interpreted as a uniform prevalence applicable across healthcare systems, particularly given the marked differences between developed and developing countries. A logit-transformed single-rate meta-analysis of all 41 included studies estimated the prevalence of ENI among critically ill patients. The pooled OR for ENI was 3.82 (95% CI: 2.52–5.78), corresponding to an overall estimated prevalence of approximately 79.2%. Extreme and largely unexplained heterogeneity was present (*I*^2^ = 99%, *p* < 0.00001). Subgroup analyses only partially explained the sources of heterogeneity. The observed variability among studies likely reflects fundamental differences in case mix, definitions of ENI, clinical practice, and measurement methods, which cannot be adequately overcome by meta-analytic pooling (Figure 2). Readers should interpret this global pooled proportion with extreme caution, and it cannot be generalized to all critical care environments.

**Figure 2:**
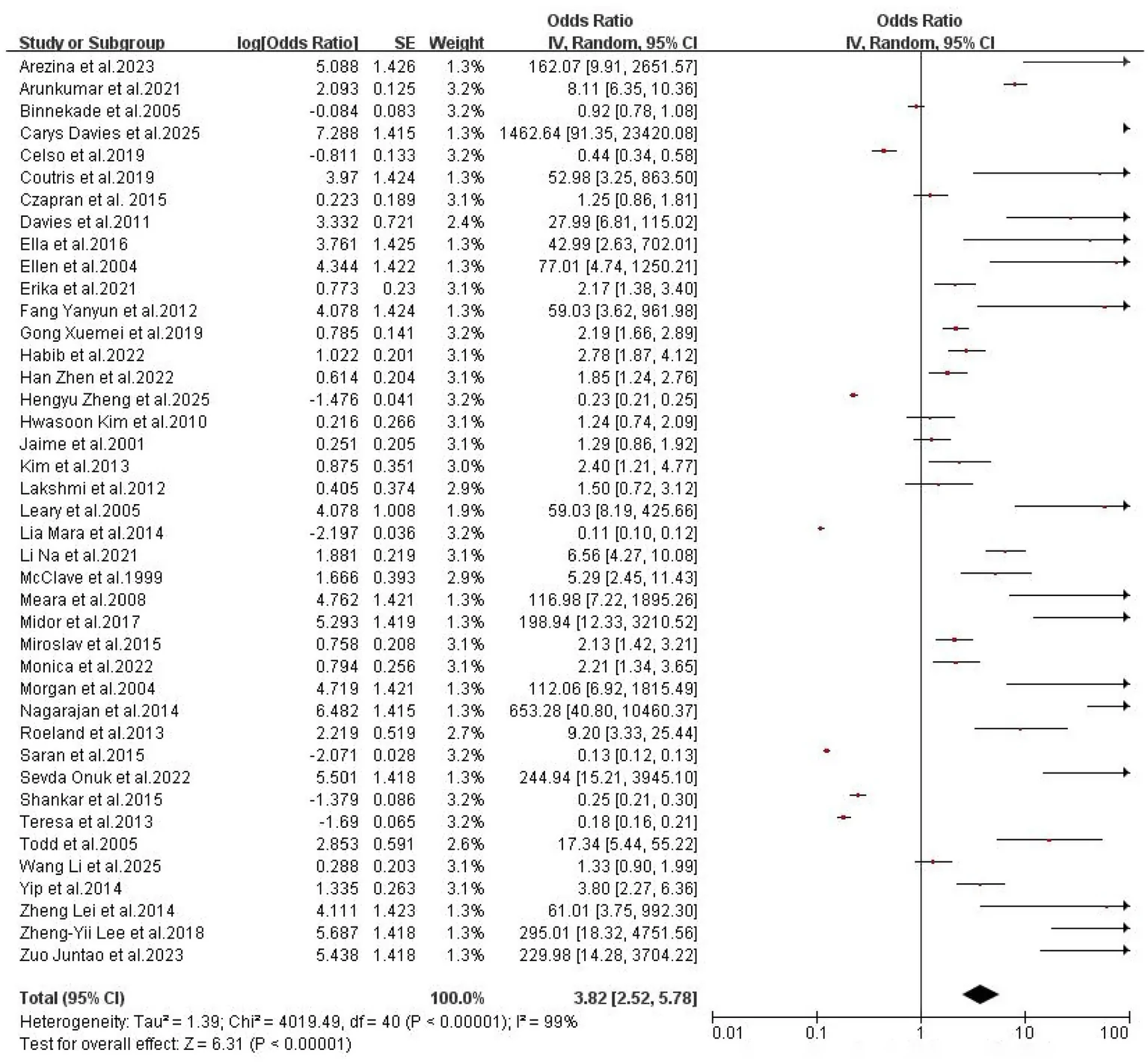
Forest plot of the pooled prevalence of ENI.

Subgroup analysis by study design revealed that the pooled OR was 5.05 (95% CI: 3.20–7.98), with extremely high heterogeneity (*I*^2^ = 99%, *p* < 0.00001). Specifically, RCTs (2 studies) showed a non-significant OR of 0.54 (95% CI: 0.10–2.91, *p* = 0.47, *I*^2^ = 99%), cross-sectional studies (4 studies) had an OR of 4.39 (95% CI: 2.11–9.11, *p* < 0.00001, *I*^2^ = 87%), prospective observational studies (29 studies) had an OR of 5.65 (95% CI: 3.02–10.56, *p* < 0.00001, I^2^ = 99%), and retrospective observational studies (7 studies) had an OR of 11.69 (95% CI: 3.83–35.69, *p* < 0.00001, *I*^2^ = 93%). The test for subgroup differences was statistically significant (*χ*^2^ = 9.18, df = 3, *p* = 0.03, *I*^2^ = 67.3%), confirming that study design was a major contributor to heterogeneity (Figure 3).

**Figure 3:**
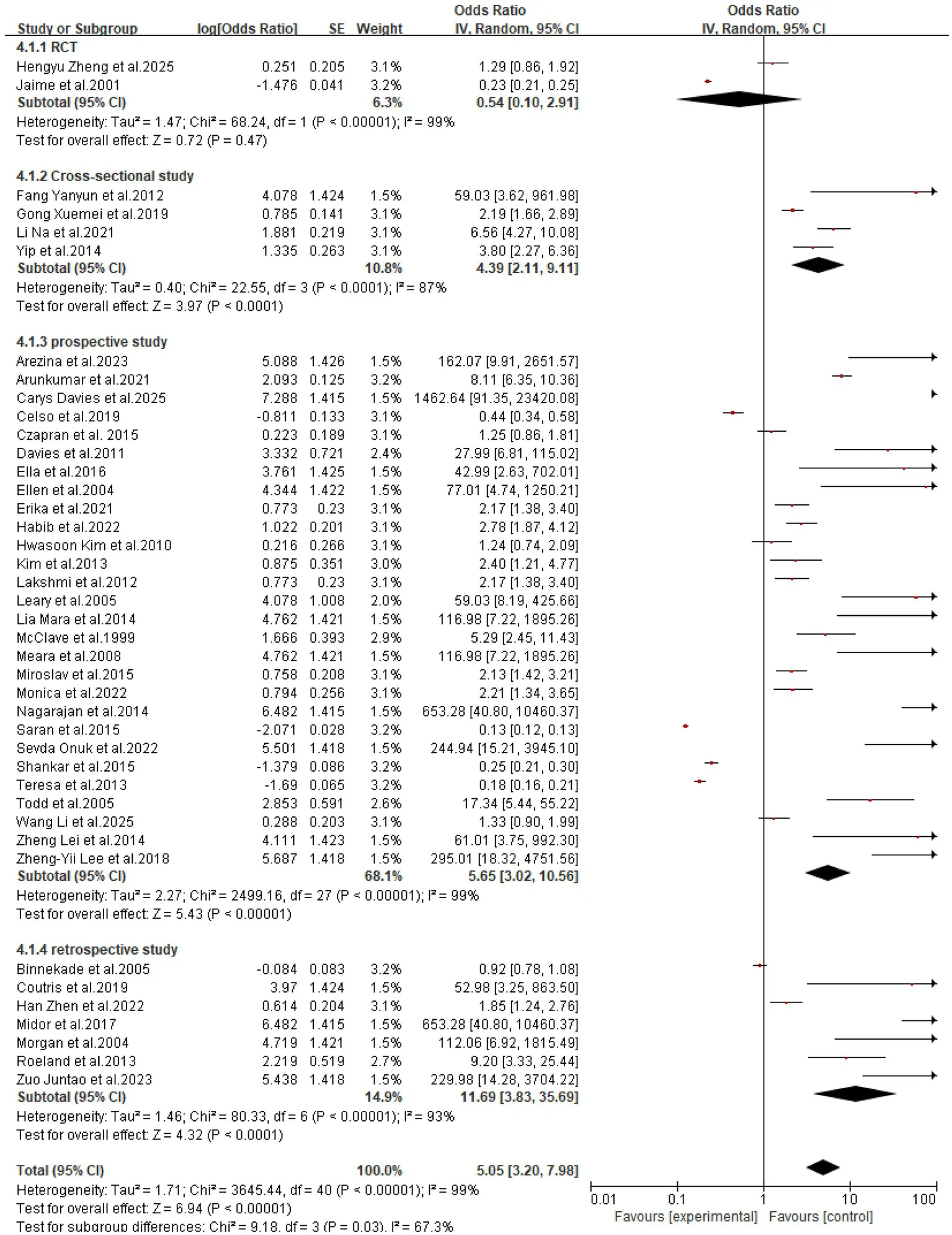
Subgroup analysis by study design.

Subgroup analysis by country development level indicated that studies from developed countries (21 studies) had an OR of 8.38 (95% CI: 4.12–17.06, *p* < 0.00001, *I*^2^ = 97%), studies from developing countries (17 studies) had an OR of 2.83 (95% CI: 1.39–5.74, *p* = 0.004, *I*^2^ = 99%), and international multicenter studies (2 studies) had a non-significant OR of 0.39 (95% CI: 0.04–3.73, *p* = 0.42, *I*^2^ = 99%). The test for subgroup differences was statistically significant (*χ*^2^ = 9.08, df = 2, *p* = 0.01, *I*^2^ = 78%), confirming that national level is a major source of heterogeneity (Figure 4).

**Figure 4:**
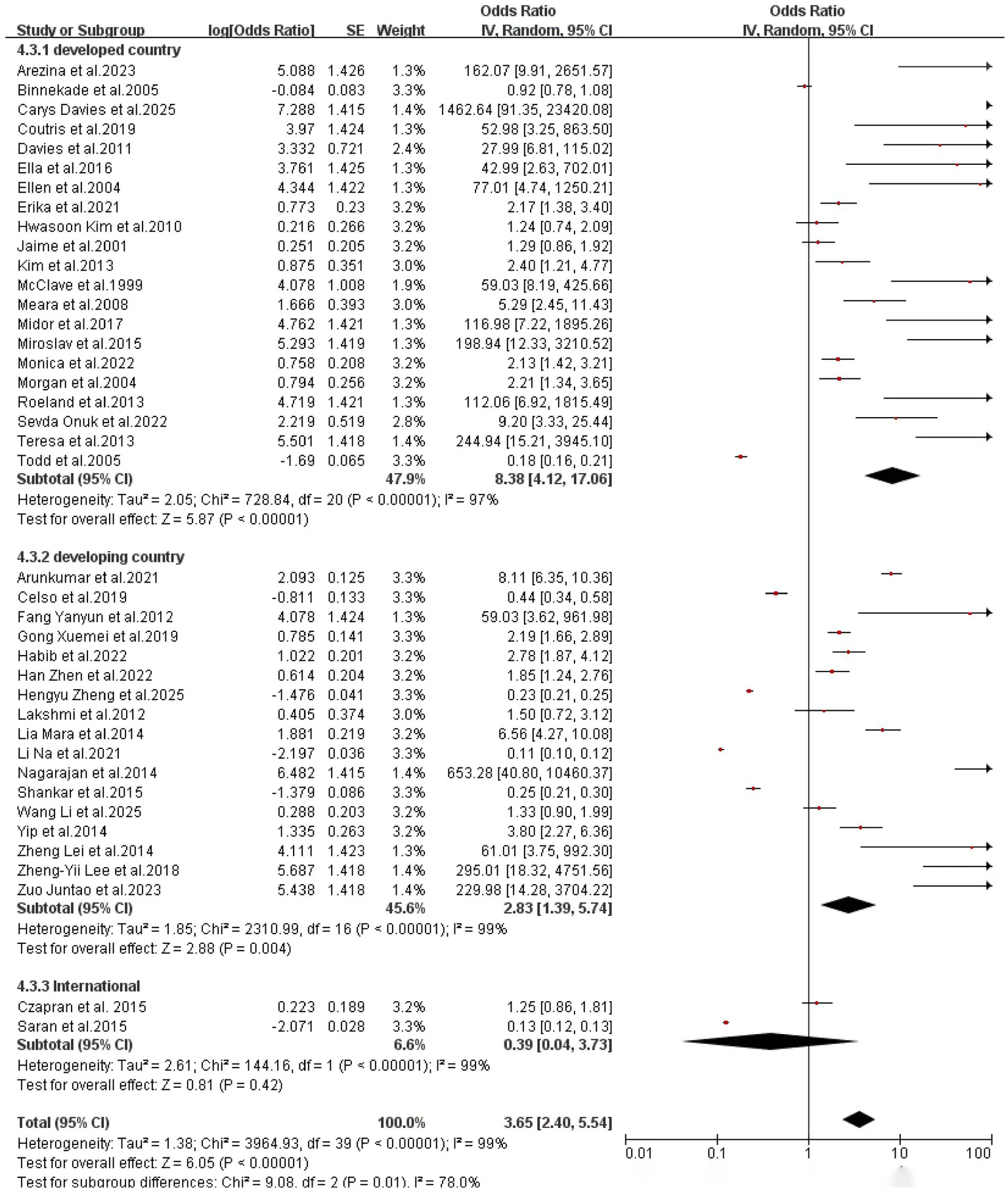
Subgroup analysis by country development level.

Subgroup analysis by enteral feeding route showed that gastric/intestinal mixed feeding studies (16 studies) had an OR of 2.18 (95% CI: 1.26–3.78, *p* = 0.005, *I*^2^ = 99%), gastric-only feeding studies (18 studies) had an OR of 7.32 (95% CI: 2.26–23.74, *p* = 0.0009, *I*^2^ = 99%), and studies without reported feeding route (7 studies) had an OR of 5.68 (95% CI: 1.99–16.23, *p* = 0.001, *I*^2^ = 95%). The test for subgroup differences was not statistically significant (*χ*^2^ = 4.89, df = 2, *p* = 0.09, *I*^2^ = 59.1%), there was insufficient statistical evidence to conclude that feeding route explained the observed heterogeneity, although a borderline trend was noted (*p* = 0.09) (Figure 5).

**Figure 5:**
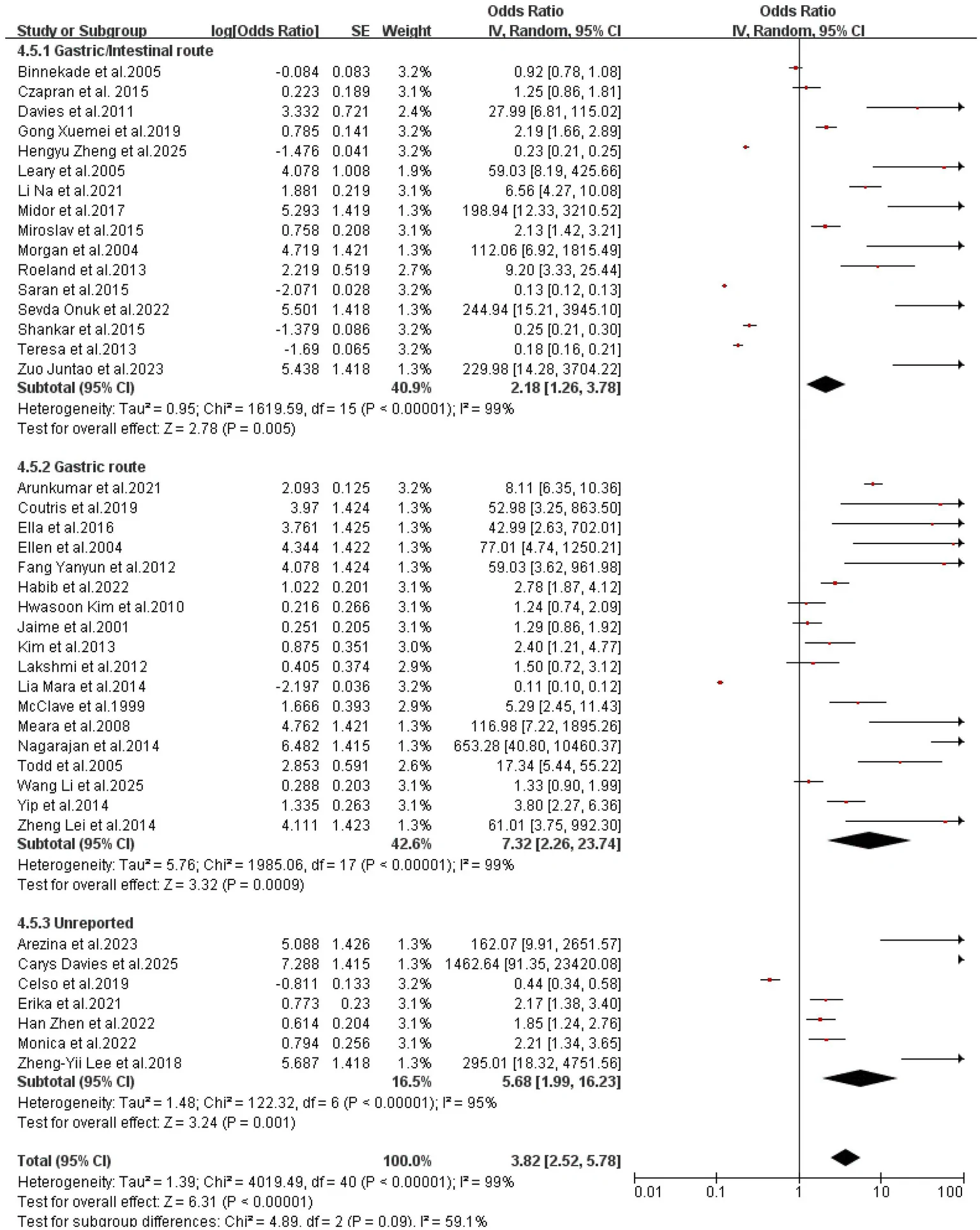
Subgroup analysis by enteral feeding route.

As clearly listed in the Definition column of Table 1b, operational time thresholds for ENI differed substantially across 41 included studies. The number of studies, total sample and crude prevalence range within each stratum are summarized in Supplementary Table S11. Thirty-two studies failed to report a standardized minimal interruption duration; only three adopted *a* ≥ 10 min threshold, three used *a* ≥ 15 min cut-off, and three applied strict standards of ≥30 min or ≥1 h. Crude ENI prevalence varied markedly across strata, the subgroup without unified definitions ranged from 11.2 to 100%, while studies with strict ≥30 min criteria reported a minimum incidence as low as 18.6%, which clearly demonstrates that the stringency of ENI definition directly alters the detected frequency of ENI. Subgroup analyses based on clinically meaningful ENI durations yielded numerically different pooled effects without statistical intergroup differences (*χ*^2^ = 3.55, df = 3, *p* = 0.31, *I*^2^ = 15.4%). This indicates inconsistent duration criteria only explain a small portion of the extreme overall heterogeneity (*I*^2^ = 99%), with patient case mix and institutional protocols as major confounders. Similarly, subgroup analyses stratified by ICU type and feeding mode also failed to resolve substantial between-study heterogeneity (Supplementary Figures S1–S3). The global pooled prevalence of 79.2% is subject to definitional bias and should be interpreted with reference to duration subgroups rather than alone.

**Table 1b:** Details of enteral nutrition interruption (ENI) and proposed interventions.

Study	Incidence (%)	Definition	Feeding route	Feeding mode	Biochemical markers	Causes of ENI	Recommended interventions
Kasti et al. (32)	100	Unreported	Unreported	Unreported	/	1. Diagnostic procedures (100%, median 3 h/d): Mainly high GRV monitoring (100%, median 3 h/d);	1. Develop interruption-minimizing protocols to reduce missed feeding hours
						2. Interventional procedures (52%, median 3.1 h/d): mainly surgery (36%, median 2.8 h/d); 3. Patient-related (25%, median 1.2 h/d): mainly GI dysfunctions (9%)	2. Involve dietitians in the multidisciplinary team for daily nutritional assessment
							3. Standardize GRV monitoring practices to avoid unnecessary ENI
							4. Implement targeted micronutrient supplementation to address deficits
Arunkumar et al. (41)	89.07	Unreported	Gastric route	Continuous infusion	Serum albumin, serum lactate	Weaning & extubation (49.7%), tracheostomy (9.2%), bronchoalveolar lavage (9.3%), Imaging procedures (8.9%), GI intolerance (15.0%)	1. Define lower pre-procedure fasting targets
							2. Restart EN promptly post-interruption
							3. Use calorie-dense formulas for fluid-restricted patients
Binnekade et al. (24)	48	Unreported	Gastric/intestinal route	Progressive continuous infusion	/	Gastric retention, feeding tube type	1. Increase energy and protein density of formulas to improve nutritional yield
							2. Standardize feeding tube use to avoid improper purposes (e.g., contrast delivery)
							3. Optimize management of feeding intolerance (e.g., rational use of prokinetic drugs)
							4. Conduct meticulous recording of intake and interfering factors to identify practice inadequacies
Davies et al. (25)	100	Unreported	Unreported	Unreported	/	1. Fasting for extubation (pre/post) (41.7%); 2. Surgical intervention (26.9%); 3. Scans (16.4%); 4. Feeding tube displacement (common in weeks 3–4); 5. GI intolerance (36.0% of patients)	1. Minimize unnecessary fasting time (e.g., optimize pre-procedural fasting protocols)
							2. Strengthen feeding tube management to reduce displacement
							3. Improve management of feeding intolerance (rational use of prokinetics)
							4. Develop trauma-specific nutrition guidelines to optimize energy and protein delivery
Ritter et al. (49)	30.77	Unreported	Unreported	Progressive continuous infusion	/	Vomiting/gastric residue (3.1%), surgery/tracheostomy (4.6%), extubation (4.6%), other/examinations (18.5%)	1. Develop and implement unit-specific standard protocols to monitor the duration of diet suspension
							2. Optimize scheduling of therapeutic procedures to reduce unnecessary fasting
							3. Strengthen multidisciplinary team collaboration to monitor and resume nutritional therapy promptly
Coutris et al. (37)	100	Unreported	Gastric route	Continuous infusion	/	Predictable (74.5%): perioperative period (main), extubation (secondary), tests/procedures (minor); unpredictable (25.5%): high GRV (main), emesis/nausea (secondary)	1. Compensate for predictable interruptions
							2. Optimize feeding schedule around procedures
							3. Use volume-based feeding goals
Czapran et al. (36)	55.56	Unreported	Gastric/intestinal route	Continuous infusion	Serum glucose	Fasting for surgery (47%), EN access loss (11%), extubation/intubation (8%), high GRV (7%), abdominal distension (7%)	1. Implement protein-energy provision via the enteral route protocol(PEP uP protocol)
							2. Start enteral nutrition at goal rate early (within 24-48 h of admission).
							3. Minimize feeding interruptions; compensate for unavoidable fasting
							4. Prefer post-pyloric over gastric feeding.
							5. Raise GRV threshold to avoid unnecessary feeding cessation.
							6. Administer gastrokinetic agents early (day 1)
							7. Consider glutamine supplementation (mean 29 g/day).
							8. Use computer-aided tools for nutrition management
Davies et al. (43)	96.55	Unreported	Gastric/intestinal route	Continuous infusion	Serum amylase, serum lipase, C-reactive protein, blood glucose	Procedure outside ICU (16%), feeding tube dislodgement (15%), vomiting (8%), planned extubation (7%), procedure in ICU (5%), high GRV (4%), abdominal distension (3%), diarrhea (1%), aspiration (0.3%)	1. Disseminate clinical practice guidelines to promote early EN
							2. Standardize nutritional therapy protocols for acute pancreatitis
							3. Reduce clinician preference-driven PN use
Segaran et al. (44)	100	Unreported	Gastric route	Continuous infusion	Blood glucose	Imaging transfer (main cause), awaiting nasogastric tube insertion (secondary cause), fasting for airway procedures (tracheostomy/extubation) (key cause), surgery (important cause)	1. Develop ICU-specific fasting guidelines (no fasting for non-airway surgery/scans; 4 h fasting for airway procedures)
							2. Provide focused education to medical/nursing staff on guidelines
							3. Reinforce guideline adherence via daily ward rounds
							4. Collaborate with anaesthetic department for protocol ratification
Elpern et al. (45)	100	Unreported	Gastric route	Progressive continuous infusion	/	Tests and procedures (35.7%), body position change (15%), unstable clinical conditions (13.5%), high GRV (11.5%), nausea & vomiting (9.2%), medication administration (7.7%), temporary EN access loss (3.4%), aspiration (2.1%), others (4.9%)	1. Establish and implement feeding protocols to standardize practices
							2. Recalculate infusion rates considering projected “down time” (e.g., 18-h infusion instead of 24-h)
							3. Limit infusion to periods with low risk of off-unit tests/procedures
							4. Avoid unnecessary interruptions for transient body position changes
Erika et al. (6)	68.49	Unreported	Unreported	Unreported	/	1. Patient-related (59%): mainly hemodynamic instability (20%), High GRV (17%), diarrhea (10%); 2. Interventional procedures (32%): mainly tracheostomy (16%), other surgeries (16%); 3. Diagnostic procedures (9%): mainly CT (6%), endoscopies (0.8% each)	1. Develop internationally recognized safe reduced-fasting guidelines for critically ill patients
							2. Implement protocols advocating for shorter peri-procedural fasting times
							3. Adopt volume-based feeding and “catching up” strategies after ENI
							4. Involve ICU dietitians in nutrition management
Fang (15)	100	Unreported	Gastric route	Continuous infusion	/	1. Difficult intubation (consciousness disturbance/bulbar palsy/improper fixation); 2. EN complications: Gastric retention (weak GI/mechanical ventilation), diarrhea (30%), hyperglycemia (30%), gastric tube blockage (6.7%); 3. Others: medical-nursing operations/fasting exams/economy/busy night shifts	1. Improve the success rate of one-time intubation: Adopt appropriate intubation methods (jaw lifting method, lateral position tongue pulling method, etc.) and strengthen fixation
							2. Prevent and manage EN complications:
							– Gastric retention: use enteral nutrition pump for continuous infusion, keep the head of the bed elevated after feeding
							– Diarrhea: select appropriate nutrient solution, control temperature and infusion speed, prevent contamination
							– Hyperglycemia: regularly monitor blood glucose and adjust nutrient solution or use insulin
							– Gastric tube blockage: rinse the tube regularly, grind drugs before injection
							3. Strengthen EN management: conduct professional training for nurses, reasonably arrange operations, communicate with family members to solve economic difficulties
Gong (14)	68.5	≥15 min	Gastric/intestinal route	Continuous infusion	/	Medical-nursing operations, difficult intubation, GI intolerance	1. Use enteral nutrition tolerance assessment and management tools to grade feeding intolerance symptoms and conduct regular active assessment
							2. Monitor the changing trend of gastric residual volume in critically ill patients by ultrasound and standardize gastric residual volume monitoring
							3. Adopt nutritional risk assessment combined with individualized and targeted nutritional management processes to reduce EN-related complications
							4. Formulate and implement early EN feeding processes for critically ill patients, strengthen multidisciplinary cooperation and relevant knowledge training
Habib et al. (39)	73.58	Unreported	Gastric route	Continuous infusion	/	BiPAP dependency (24.5%), tracheostomy (21.7%), preoperative fasting (16%), dialysis (17.9%)	1. Optimize BiPAP use to reduce interruptions
							2. Minimize pre-surgical NPO duration
							3. Standardize tracheostomy-related feeding management
Han et al. (11)	64.84	≥10 min	Unreported	Unreported	Albumin, total protein, hemoglobin	Medical-nursing operations, diagnostic exams, pipeline factors, GI bleeding, hemodynamic instability, physical rehabilitation, dialysis, position change, equipment failure/delay	1. Avoid excessive use of analgesic and sedative drugs, dynamically assess the level of analgesia and sedation, and adopt a goal-directed analgesia and sedation strategy
							2. Improve feeding tolerance management, use enteral nutrition tolerance assessment and management tools, and conduct hierarchical treatment of feeding intolerance
							3. Establish an enteral nutrition checklist, train nurses on relevant knowledge and supervise the implementation, and improve the nutrition process
							4. Establish a prevention and management strategy for ENI in ICU patients to reduce ENI caused by subjective factors
							5. Provide practical ENI handling procedures to correctly respond to interruption events
Zheng et al. (21)	18.63	≥30 min	Gastric/intestinal route	Continuous infusion	/	1. Diagnostic & therapeutic procedures (50.9%); 2. Others: EN tube dysfunction/hemodynamic instability/anastomotic leakage/rehabilitation/patient refusal (26.8%); 3. GI intolerance (high GRV/abdominal distension/vomiting/diarrhea) (22.3%)	1. Establish comprehensive nutrition support protocols for critical care nurses
							2. Strengthen training for department nurses to equip them with skills for preventing and managing ENI
							3. Standardize EN management protocols to avoid unnecessary interruptions
							4. Develop dynamic feeding algorithms integrating real-time gastrointestinal tolerance assessment and critical care treatment phases
Kim et al. (19)	55.32	Unreported	Gastric route	Intermittent feeding	Prealbumin, transferrin; CRP	Extubation/intubation (34.6%), GI bleeding (23.1%), High GRV (15.4%), condition deterioration (11.5%), external drainage tube insertion (7.7%), cerebral angiography (7.7%), others (3.8% each)	1. Develop evidence-based and safe clinical practice guidelines for enteral feeding management
							2. Standardize the judgment criteria for stopping/delaying feeding (e.g., rationalize gastric residual volume cutoff values)
							3. Avoid overprotective feeding management and reduce avoidable interruptions
							4. Nurses should strictly follow standardized protocols to improve the adequacy of enteral feeding
Pinilla et al. (22)	56.25	Unreported	Gastric route	Continuous infusion	Serum albumin, serum magnesium	1. GI intolerance: high GRV (Group I 53%/Group II 23%, *p* < 0.005), Vomiting (Group I 6%/Group II 7%), Diarrhea (Group I 11%/Group II 23%); 2. Others: Surgery (Group I 6/Group II 10), extubation (Group I 6/Group II 5), physiotherapy (3 each), EN tube issues/hypophosphatemia	1. Adopt higher gastric residual volume threshold (250 mL vs. 150 mL)
							2. Mandatory prokinetic administration for all patients (metoclopramide, cisapride, domperidone)
							3. Standardize enteral feeding progression protocol (start at 25 mL/h, increase by 25 mL/h every 4 h if tolerated)
Kim et al. (28)	70.59	Unreported	Gastric route	Intermittent feeding	Serum albumin, C-reactive protein	GI intolerance (29.5%, *r* = −0.874), routine care (1.4%), procedures (17.5%, *r* = −0.839), airway management (22.4%), GI bleeding (18.9%), EN tube issues (1.2%)	1. Develop standardized enteral feeding protocols
							2. Provide continuing education for healthcare providers on nutritional support
							3. Optimize GRV cutoff (current 50 mL may be too low)
							4. Minimize fasting time before procedures
							5. Flexible feeding schedule for intermittent bolus feeding
Lakshmi and Vaidyanathan (40)	60	Unreported	Gastric route	Unreported	/	Airway management (28%), surgical intervention (20%), GI intolerance (vomiting/diarrhea) (12%)	1. Implement tube feeding protocols to standardize practices and limit unnecessary interruptions
							2. Strengthen coordination between multidisciplinary teams to reduce procedural interruptions
							3. Optimize management of GI intolerance to minimize feeding cessation
O’Leary-Kelley et al. (42)	98.33	≥15 min	Gastric/intestinal route	Continuous infusion	/	1. ICU-related (65.1%): extubation/airway management (28.8%), preoperative fasting (23.4%), exam fasting (6.9%), etc.; 2. GI factors (19.8%): Vomiting (6.8%), high GRV (6.6%), etc.; 3. Tube factors (11.1%): dislodgement/clogging (4.6%/4.2% each)	1. Develop standardized enteral feeding protocols to limit unnecessary interruptions
							2. Strengthen monitoring of daily nutritional intake by nursing staff
							3. Advocate for early initiation of enteral feeding
							4. Optimize timing of procedures to minimize feeding interruption duration
Li (17)	86.75	Unreported	Gastric/intestinal route	Continuous infusion	Serum albumin, white blood cell count	GI symptoms (47.59%), surgery (6.63%), exams (1.20%), rescue (0.60%), rehabilitation exercise (1.81%)	1. Establish nutrition support teams and develop standardized EN protocols
							2. Conduct EN knowledge training for ICU doctors and nurses
							3. Avoid unnecessary EN interruption; relieve gastrointestinal symptoms before suspending EN
							4. Monitor serum albumin levels and supplement albumin if necessary
							5. Adjust PEEP for mechanically ventilated patients and discontinue ventilation early
Ribeiro et al. (18)	100	Unreported	Gastric route	Progressive continuous infusion	CRP	1. Internal (78.1%): extubation (29.9%), GI complications (21.4, 70% from high GRV), EN tube issues (17.9%), routine causes (8.9%); 2. External (21.9%): Tracheostomy (9.5%), endoscopy (5.5%), tomography (5.3%)	1. Optimize the management of internal causes of ICU, mobilize the entire medical team to control interruptions related to extubation, gastrointestinal complications and feeding tubes
							2. Establish flowcharts to improve the logistics coordination with other hospital departments, reducing external cause interruptions
							3. Strengthen the monitoring and intervention of patients with acute inflammatory response (elevated CRP) to improve nutritional tolerance
							4. Pay attention to the nutritional support of elderly patients (>60 years old) and reduce the impact of dysphagia on oral route recovery
McClave et al. (29)	84.09	Unreported	Gastric route	Continuous infusion	Serum albumin	High GRV (45% patients, 15.14% duration), EN tube displacement (41% patients, 7.66% duration), procedures (39% patients, 34.99% duration), diagnostic exams (27% patients, 4.62% duration), others (32% patients, 36.15% duration)	1. Implement strict enteral feeding infusion protocols
							2. Educate healthcare professionals on avoidable cessation reasons
							3. Rationalize GRV management (200 mL as reasonable cutoff)
							4. Shorten fasting time before procedures (up to 4 h before)
							5. Use high-calorie dense formulas
							6. Aggressive tube fixation to prevent displacement
O’Meara et al. (30)	100	≥15 min	Gastric route	Continuous infusion	/	Small-bore EN tube issues (25.6%), High GRV (13.3%), weaning (11.7%), procedures (7.9%), surgery preparation (7.7%)	1. Optimize small-bore feeding tube management
							2. Rethink residual volume monitoring thresholds
							3. Minimize unnecessary interruptions for procedures/nursing care
Uozumi et al. (38)	100	Unreported	Gastric/intestinal route	Progressive continuous infusion	/	Undetermined causes (29%), airway manipulation (18%), GI events (abdominal pain/high GRV/vomiting/diarrhea/GI bleeding) (14%), dialysis (13%), ICU diagnostic-therapeutic procedures (11%), others (surgery/tracheostomy, etc.)	1. Develop a standardized protocol for EN interruption and resumption
							2. Shorten unnecessary pre-procedural fasting time (e.g., follow 6-h fasting consensus for tracheostomy)
							3. Improve staff awareness of calorie deficits caused by ENI
							4. Document clear orders for EN interruption and resumption
Peev et al. (20)	68.09	≥10 min	Gastric/intestinal route	Progressive continuous infusion	Serum albumin, prealbumin, C-reactive protein	Extubation/intubation (27.36%), Tracheostomy/PEG (21.70%), imaging exams (15.09, 87.5% avoidable), ortho procedures (11.32, 50% avoidable), High GRV (9.43%), others (5.66% each, 66.7% avoidable)	1. Maximize nutrient delivery before and after unavoidable EN interruptions
							2. Adopt “PEP uP Protocol” (higher initial EN rate, prophylactic prokinetics, catch-up boluses)
							3. Standardize periprocedural EN criteria
Van Nieuwkoop et al. (27)	68.85	Unreported	Unreported	Unreported	/	CT scan (23.5%), High GRV (16.5%), Nasogastric tube dysfunction (13.9%), Planned extubation (8.7%), Surgery (6.1%), Intubation (6.1%), others (MRI/bronchoscopy, etc.)	1. Incorporate ENI considerations into ICU nutritional protocols
							2. Optimize diagnostic procedure scheduling to reduce unplanned ENI
							3. Strengthen nasogastric tube management to minimize dysfunction
							4. Adopt reduced fasting protocols to shorten interruption duration
Morgan et al. (33)	100	Unreported	Gastric/intestinal route	Continuous infusion	Serum albumin, serum prealbumin	Surgery (27%), diagnostic procedures (15%), GI intolerance (11%), EN tube issues (8%)	1. Precise timing for stopping/restarting EN before/after procedures
							2. Increase jejunostomy/nasojejunal tube use
							3. Aggressive prokinetic use for GI intolerance
Daphnee et al. (23)	100	Unreported	Gastric route	Continuous infusion	/	1. In-CCU procedures (55.9%): Ventilation-related (40.25%), bedside procedures (5.48%), etc.; 2. GI symptoms (24.03%): Nasogastric aspiration (15.28%), diarrhea/vomiting (3.50%), etc.; 3. Out-of-CCU procedures (18.31%): Surgery (7.11%), radiology (6.76%), etc.; 4. Others (1.86%)	1. Modify feeding protocols to reduce avoidable ENI (especially for procedures inside CCU)
							2. Incorporate strategies to avoid or shorten ENI duration for CCU procedures into clinical practice guidelines
							3. Standardize the management of GI symptoms during EN to minimize prolonged interruptions
							4. Strengthen coordination for procedures outside CCU to reduce unnecessary feeding cessation
Passier et al. (46)	90.24	Unreported	Gastric/intestinal route	Unreported	/	Planned procedure-related (94.4%), unplanned procedure-related (5.6%), ICU departure only (11.1%), unnecessary interruption (23%), no documented order (31%)	1. Adhere to guidelines promoting short cessation periods
							2. Clarify which procedures truly require EN cessation via interdisciplinary agreement
							3. Eliminate the “fast from midnight” approach; restart EN promptly at pre-cessation rate after procedures
							4. Standardize documentation of EN cessation orders
							5. Minimize postponed or cancelled procedures
Saran et al. (35)	11.24	Unreported	Gastric/intestinal route	Continuous infusion	/	High GRV (main cause), abdominal distension (secondary cause), vomiting (secondary cause)	1. Use bedside feeding protocols
							2. Optimize HOB elevation
							3. Early EN initiation
Onuk et al. (26)	100	Unreported	Gastric/intestinal route	Unreported		Radiological procedures (27.2%), EN tube malfunction (pull-out/clogging/reinsertion) (14.7%), surgical procedures (11.1%), hemodynamic instability (9.6%), airway procedures (6.3%), high GRV (>300 mL) (5.7%), others (vomiting/GI bleeding, etc.)	1. Optimize radiological procedure scheduling to reduce ENI frequency
							2. Strengthen feeding tube fixation and maintenance to prevent malfunctions
							3. Standardize management of high gastric residual volume and feeding intolerance
							4. Develop protocols to minimize ENI duration and compensate for nutritional deficits
Shankar et al. (34)	20.13	Unreported	Gastric/intestinal route	Continuous infusion	/	High GRV (>250 mL) (14.6%), GI bleeding (2.3%), Gastroparesis (2.3%), Nausea/vomiting (2.3%)	1. Routine initiate EN within 6 h of ICU admission
							2. Strengthen daily monitoring of calorie/protein deficits
							3. Standardize EN interruption criteria
Williams et al. (48)	15.45	Unreported	Gastric/intestinal route	Continuous infusion	/	907 episodes pre-intervention vs. 662 post: extubation prep/airway management (38.81% → 41.39%), procedure-related (25.47% → 28.70%), GI issues (14.22% → 9.82%, *p* < 0.01), EN delivery system issues (8.49% → 7.85%)	1. Develop revised EN flow diagram for GRV management 2. Train EN Champions to promote protocol adherence 3. Provide formal and informal education on reducing unnecessary interruptions 4. Avoid fasting for procedures unless necessary
Rice et al. (50)	94.55	Unreported	Gastric route	Continuous infusion	/	Procedures (bedside/OR) (41%), no documented reason (21%), planned extubation (15%), hemodynamic instability/shock (6%), GI intolerance (9%), EN tube issues (3%), others (bath/transfer, 2% each)	1. Implement standardized enteral feeding protocols to promote early initiation and rapid advancement to target rates
							2. Standardize management of gastrointestinal intolerances (e.g., tolerate gastric residuals up to 300 mL before interruption)
							3. Improve documentation of reasons for feeding interruptions
							4. Train medical staff on protocol implementation to ensure consistent practice
Wang and Li (16)	57.14	Continuous pumping: Interruption time > 1 h; Intermittent injection: 3 times daily, 30 min each time, but failed to obtain expected nutrition within 30 min	Gastric route	Continuous infusion	/	Independent risk factors (OR): Mechanical ventilation (synchronized intermittent) (2.305), acid suppressant use (2.875), antibiotics ≥2 types (2.732), mild hypothermia (2.703)	/
Yip et al. (31)	79.22	≥1 h	Gastric route	Continuous infusion	/	Procedures (45.1%), High GRV (38.0%), Diarrhea (8.4%), Nasogastric tube placement difficulty (5.6%)	1. Develop standardized enteral feeding protocols
							2. Rationalize GRV management
							3. Shorten fasting time before procedures
Zheng et al. (13)	100	Unreported	Gastric route	Continuous infusion	/	Abdominal distension (38.1%), gastric retention (26.4%), medical procedures/exams (17.3%), vomiting (11.4%), GI bleeding (9.2%), EN tube dysfunction (5.8%), hemodynamic instability (4.3%), abdominal pain (1.4%)	1. Pay close attention to the observation of gastrointestinal tolerance in patients using norepinephrine, propofol, and remifentanil
							2. Take preventive measures to avoid complications such as aspiration pneumonia
							3. Consider adding parenteral nutrition therapy based on the patient’s specific clinical conditions
Lee et al. (47)	100	Unreported	Unreported	Intermittent feeding		Respiratory procedures (27.1%), planned procedure not executed post-fasting (16.9%), unknown reason (12.7%), radiological procedures (11.4%), inappropriate reasons (7.8%), GI bleeding (6.9%), others (OR/bedside procedures, etc.)	1. Implement evidence-based feeding protocols supervised by a nutrition support team
							2. Revise feeding protocols based on latest research findings
							3. Provide regular staff training on feeding guidelines
							4. Minimize avoidable interruptions (e.g., standardize fasting time before procedures)
Zuo et al. (12)	100	≥10 min	Gastric/intestinal route	Continuous infusion	/	Out-of-ward exams (CT/MRI) (33%), GI intolerance (15%), medical procedures (13%), unknown causes (11%), basic care (10%), pipeline factors (6%), airway operations (4%), CRRT-related (4%), surgery/prep (3%)	1. Proactively use enteral nutrition tolerance assessment and management tools, and conduct hierarchical treatment for feeding intolerance symptoms
							2. Establish an evidence-based ENI prevention and management plan through group discussions and expert consultations
							3. Adopt volume-based feeding (VBF) protocol: Adjust the infusion rate according to remaining time and intake when restarting EN after interruption
							4. Strengthen multidisciplinary cooperation and nutrition knowledge training to improve medical staff’s cognition of ENI
							5. Formulate targeted training goals for nursing staff at different levels to enhance their ENI management awareness and execution ability
							6. Resume EN in a timely manner after clinical operations to avoid prolonged interruption

To further verify the stability of prevalence estimates restricted to clinically meaningful sustained feeding holds, we performed stratified leave-one-out sensitivity analyses separately for three groups with explicit ENI duration cut-offs (Supplementary Tables S12–S14). For the mild clinical threshold (ENI ≥ 10 min), the random-effects pooled OR remained statistically significant (OR = 2.93, 95%CI 1.23–6.99, *p* = 0.02). After removing the outlier study within this subgroup, between-subgroup heterogeneity was eliminated (*I*^2^ = 0%), and consistent significant pooled effects were observed under both fixed- and random-effects models. For the moderate threshold (ENI ≥ 15 min), the pooled OR remained significantly elevated (OR = 20.04, 95%CI 1.13–353.94, *p* = 0.04); exclusion of the outlier study fully eliminated subgroup heterogeneity without altering the positive association. For the strict long-duration definition (ENI ≥ 30 min, representing prolonged interruptions linked to substantial caloric deficits), the pooled effect turned non-significant (OR = 1.04, 95%CI 0.18–6.05, *p* = 0.97), which was largely attributable to the limited number of eligible studies in this stratum. Collectively, stratified sensitivity analyses focusing on clinically meaningful ENI durations further confirmed that the high overall pooled prevalence of 79.2% was largely driven by studies counting all trivial brief feeding pauses without standardized time thresholds.

Sensitivity analysis using a leave-one-out approach confirmed the robustness of the results, with no single study materially influencing the pooled estimate (Supplementary Figure S4). Funnel plot asymmetry suggested potential publication bias and small-study effects (Supplementary Figure S5). This was supported by Egger’s regression test (intercept = 9.29, SE = 1.26, *p* < 0.001), indicating significant asymmetry. Consistently, the Egger’s regression plot showed clear deviation from the horizontal axis (Supplementary Figure S6). Together, these findings suggest possible publication bias and a potential overestimation of the pooled effect size.

### Reasons for ENI

In this study, the reasons for ENI were categorized into nine main groups: airway management procedures, bedside diagnostic/therapeutic procedures, medical care procedures, gastrointestinal factors, hemodynamic factors, device-related problems, radiological procedures, surgical procedures/preparation, and other or undocumented causes (Table 2). The most frequent causes were radiological procedures, airway management, and surgical procedures. Radiological procedures were reported in 12 studies, with a median incidence of 15.0% (IQR, 8.8–27.4%), airway management in 11 studies had a median of 27.1% (IQR, 16.2–43.4%), and surgical procedures or preoperative preparation in 10 studies had a median of 10.4% (IQR, 4.9–19.6%).

**Table 2:** Causes of ENI in included studies.

Reasons for interruption									
Study	①	②	③	④	⑤	⑥	⑦	⑧	⑨
Arunkumar et al. (41)	615 (66.3%)	86 (9.3%)	–	139 (15.0%)	–	–	83 (8.9%)	–	–
Davies et al. (25)	780 (41.7%)	–	–	481 (36.0%)	–	–	307 (16.4%)	504 (26.9%)	–
Erika et al. (6)	22 (16.79%)	–	–	46 (35.11%)	26 (19.85%)	4 (3.05%)	11 (8.39%)	21 (16.03%)	1 (0.76%)
Zheng et al. (21)	–	–	–	157 (22.3%)	–	–	359 (50.9%)	–	189 (26.8%)
Uozumi et al. (38)	125 (22%)	136 (24%)	–	79 (14%)	–	–	28 (5%)	11 (2%)	166 (29%)
Peev et al. (20)	52 (49.06%)	10 (9.43%)	–	10 (9.43%)	–	–	16 (15.09%)	20 (20.75%)	6 (5.66%)
Van Nieuwkoop et al. (27)	18 (15.65%)	1 (0.87%)	–	20 (17.39%)	1 (0.87%)	16 (13.91%)	32 (27.83%)	7 (6.09%)	16 (13.97%)
Morgan et al. (33)	–	–	–	24 (10.81%)	–	18 (8.11%)	33 (14.86%)	91 (40.99%)	56 (25.23%)
Daphnee et al. (23)	345 (40.25%)	48 (5.6%)	47 (5.48%)	206 (24.03%)	–	–	73 (8.51%)	84 (9.79%)	16 (1.86%)
Onuk et al. (26)	–	–	–	104 (31.2%)	32 (9.6%)	49 (14.7%)	91 (27.2%)	37 (11.1%)	–
Rice et al. (50)	27 (15%)	73 (41%)	–	18 (10%)	11 (6%)	5 (3%)	–	–	45 (25%)
Yip et al. (31)	32 (45.1%)	–	–	33 (46.48%)	–	4 (5.6%)	–	–	–
Lee et al. (47)	90 (27.11%)	70 (21.08%)	26 (7.83%)	26 (7.83%)	2 (0.6%)	3 (0.9%)	38 (11.45%)	15 (4.52%)	42 (12.65%)
Zuo et al. (12)	8 (6%)	5 (4%)	32 (23%)	21 (15%)	–	8 (6%)	45 (33%)	4 (3%)	15 (11%)
Studies reporting (*n*)	11	8	3	14	5	8	12	10	10
Median incidence (IQR), %	27.1 (16.2–43.4)	9.4 (5.2–21.8)	7.8 (6.7–15.4)	16.2 (11.6–29.4)	6.0 (0.9–9.6)	5.8 (3.0–9.6)	15.0 (8.8–27.4)	10.4 (4.9–19.6)	13.3 (7.0–25.2)

① Procedures during ICU stay: airway management procedures. ② Procedures during icu stay: bedside diagnostic & therapeutic procedures. ③ Procedures during icu stay: medical care procedures. ④ Patient: gastrointestinal Factors. ⑤ Patient: hemodynamic factors. ⑥ Device & technical factors: feeding tube-related problems. ⑦ Procedures outside of ICU: radiological/imaging procedures. ⑧ Procedures outside of ICU: surgical procedures & preoperative preparation. ⑨ Other & undocumented causes.

ICU, intensive care unit. Values are presented as number of events with percentages. “–” indicates that the cause was not reported in the study. Median incidence is presented as median (interquartile range, IQR) across included studies.

Other notable causes included gastrointestinal intolerance (16.2%, IQR 11.6–29.4%, *n* = 14), device-related problems (5.8%, IQR 3.0–9.6%, *n* = 8), hemodynamic factors (6.0%, IQR 0.9–9.6%, *n* = 5), and bedside diagnostic/therapeutic procedures (9.4%, IQR 5.2–21.8%, *n* = 8). Three studies reported other or undocumented causes exceeding 40% of interruptions, reflecting variability in reporting practices.

Overall, these findings indicate that procedures both inside and outside the ICU, airway management, and gastrointestinal intolerance are the predominant contributors to ENI, suggesting potential targets for interventions to reduce interruption frequency.

### Impact of ENI on clinical outcomes

Three studies (6, 20, 21) reported data on energy adequacy (Table 3). We pooled the energy adequacy rates using a fixed-effects model, as between-study heterogeneity was low (*I*^2^ = 24%; *p* = 0.27). The pooled MD was −4.21 (95% CI, −7.71 to −0.71), indicating that ENI was associated with a significantly lower energy adequacy rate compared with non-ENI (*p* = 0.02) (Figure 6A).

**Table 3:** The impact of ENI on the clinical outcomes of critically ill patients.

Outcome	Davies et al. (25)		Erika et al. (6)		Zheng et al. (21)		Peev et al. (20)	
	ENI (*n* = 732)	No ENI (*n* = 238)	ENI (*n* = 50)	No ENI (*n* = 23)	ENI (*n* = 248)	No ENI (*n* = 1,083)	ENI (*n* = 64)	No ENI (*n* = 30)
Energy adequacy, % mean ± SD	N/R	–	96 ± 22	97 ± 24	66.56 ± 28.23	70.14 ± 27.38	63 ± 27.47	76 ± 26.22
ICU length of stay, *d* median (IQR)	N/R	–	17 (10–26)	13 (8–26)	21 (10–28)	22 (12–28)	15 (10–28)	13.5 (8–23)
Short-term mortality, *n* (%)	151 (20.6)	10 (4.2)	23 (46.0)	5 (21.7)	30 (12.1)	149 (13.8)	5 (7.8)	2 (6.7)

ENI, enteral nutrition interruption; N/R, not reported; SD, standard deviation; IQR, interquartile range; CI, confidence interval.

**Figure 6:**
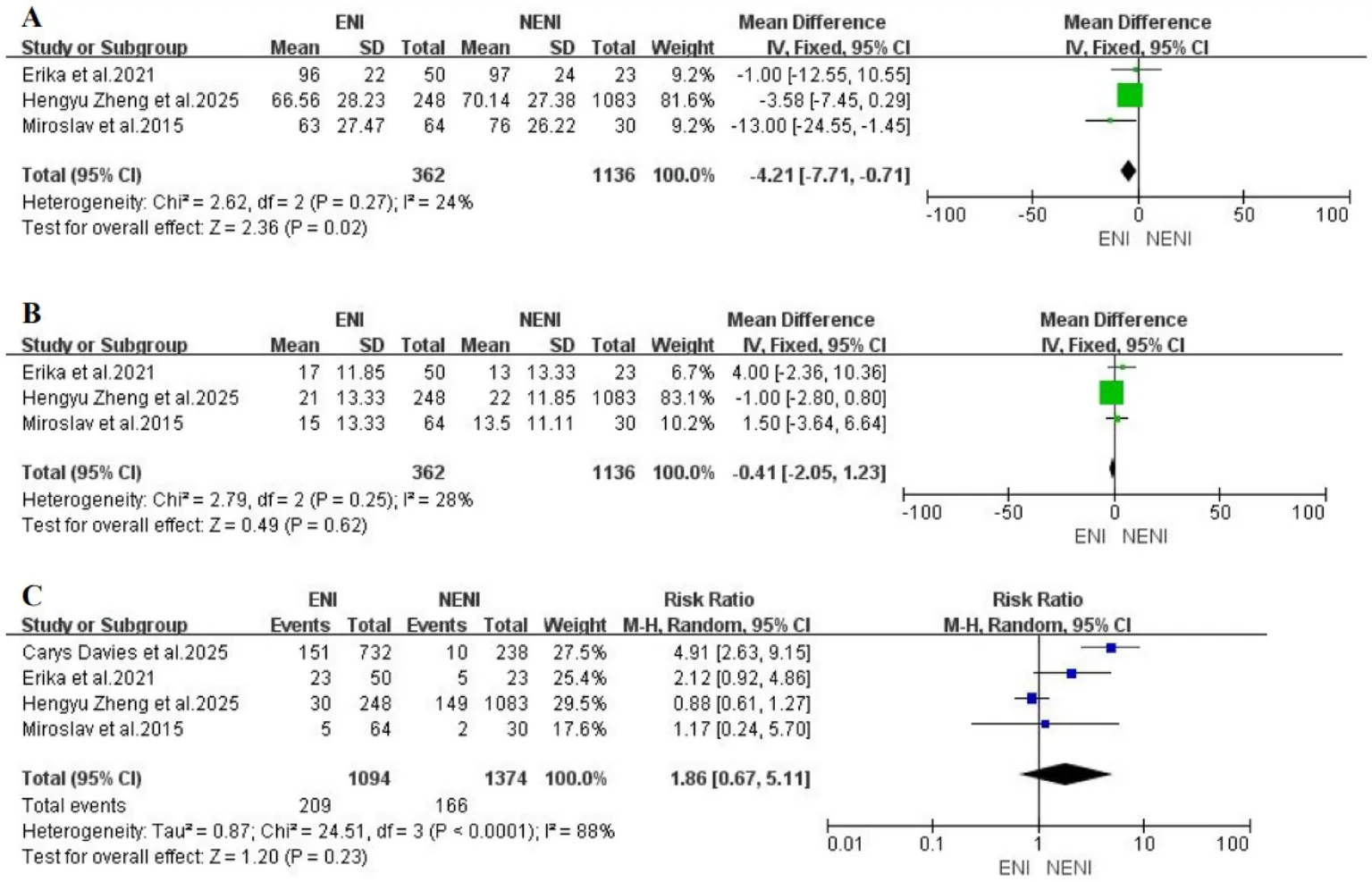
Forest plots of association between ENIs and clinical outcomes in critically ill patients. **(A)** Energy adequacy; **(B)** ICU length of stay; and **(C)** short-term mortality.

Three studies (6, 20, 21) reported ICU LOS data. Given the low between-study heterogeneity (*I*^2^ = 28%; *p* = 0.25), a fixed-effects model was applied in accordance with standard meta-analytic practice. Indicating the pooled estimate did not reach statistical significance, yet the 95% CI [−2.05, 1.23] was wide and could not exclude either prolonged or shortened ICU stay related to ENI (*p* = 0.62) (Figure 6B).

Four studies (6, 20, 21, 25) reported data on short-term mortality (including 28-day, 30-day, and in-ICU mortality). Given the substantial between-study heterogeneity (*I*^2^ = 88%; *p* < 0.0001), a random-effects model was employed in accordance with standard meta-analytic practice. Although the point estimate suggested a potential increase in mortality risk (RR = 1.86), the wide 95% CI indicated high imprecision and low statistical power. Therefore, we cannot rule out a clinically meaningful causal relationship between ENI and short-term mortality due to severe imprecision of the pooled estimate (Figure 6C). Sensitivity analysis was performed by excluding the study 26 with the most extreme effect size, which markedly reduced heterogeneity (*I*^2^ = 34%; *p* = 0.22). The updated pooled RR was 1.00 (95% CI, 0.67–1.49; *Z* = 0.00; *p* = 1.00), confirming that the primary finding of no significant association remained robust (Supplementary Figure S7).

### Certainty of evidence

GRADE assessments for primary outcomes explored in meta-analyses are presented in Table 4. Given that most included studies were observational in design, the initial certainty of evidence was rated as low. The certainty was further downgraded according to risk of bias, inconsistency, indirectness, imprecision, and publication bias. For energy adequacy, the certainty of evidence was rated as low due to moderate risk of bias and limited number of studies. For ICU length of stay, the certainty of evidence was also rated as low, mainly driven by imprecision and limited sample size. For short-term mortality, the certainty of evidence was rated as very low, due to substantial heterogeneity (*I*^2^ = 88%), serious imprecision, and evidence of publication bias. Overall, the certainty of evidence for all clinical outcomes ranged from low to very low, indicating that further high-quality studies are highly likely to change the estimates.

**Table 4:** GRADE evidence quality assessment of ENI outcomes.

Outcome	No. of studies	Study design	Risk of bias	Inconsistency (*I*^2^)	Publication bias	Imprecision	Overall certainty
Energy adequacy	3	Observational/RCT secondary	Serious	Low–moderate (24%)	Not evident	Moderate	Low
ICU length of stay	3	Observational	Serious	Low (28%)	Not evident	Serious	Low
Short-term mortality	4	Observational	Serious	High (88%)	Present	Very serious	Very low

### Qualitative results

Among the 41 included studies, 13 reported biochemical markers related to ENI (Table 1b). Eight studies (11, 17, 20, 22, 28, 29, 33, 41) all mentioned this biomarker, serum albumin, primarily reflecting nutritional status. Inflammatory markers were reported in five studies (18–20, 25, 28), most commonly C-reactive protein (CRP), sometimes combined with albumin or prealbumin. Three studies evaluated metabolic or pancreatic-related markers, including blood glucose (36, 44) and amylase/lipase (25). Other less frequently reported indicators included prealbumin (19, 20, 33), transferrin (19), white blood cell count (17), serum lactate (41), and magnesium (22). Overall, biochemical markers were inconsistently reported across studies, with no standardized measurement approaches or consistent associations with ENI identified.

Most studies also proposed interventions aimed at reducing ENI and improving nutritional delivery. These recommendations generally focused on four key aspects. First, several studies (28, 36, 50) emphasized the importance of standardized EN protocols, including clear criteria for ENI and resumption, standardized gastric residual volume thresholds, and structured feeding algorithms. Second, optimization of peri-procedural fasting management was frequently recommended, such as minimizing unnecessary fasting before diagnostic or therapeutic procedures and restarting feeding promptly after procedures (41, 43, 46). Third, improved management of gastrointestinal (GI) intolerance-induced interruptions and procedure-related operational withholding of EN was highlighted, as these two distinct root causes require differentiated targeted intervention bundles; corresponding measures include the rational use of prokinetic agents, appropriately elevated gastric residual volume thresholds, and strengthened tube fixation and maintenance (22, 26, 29). Finally, several studies advocated multidisciplinary nutritional management, including the involvement of nutrition support teams, regular staff training, and formal implementation of volume-based feeding as a core compensatory strategy to offset caloric deficits accumulated during repeated ENI (6, 12, 47). Collectively, these tiered, cause-tailored strategies aim to reduce avoidable interruptions and improve the adequacy of EN delivery in critically ill patients.

## Discussion

This systematic review and meta-analysis of 41 studies including 8,260 critically ill adult patients was conducted to determine the pooled prevalence, contributing reasons, key outcome indicators, and evidence-based interventions for ENI in the ICU.

The pooled ENI prevalence of 79.2% cannot be treated as a universal benchmark for ICU nutrition practice. Extreme inter-study heterogeneity (*I*^2^ = 99%) could only be partially explained by study design and national level. Broad discrepancies in patient populations, inconsistent ENI duration cutoffs, variable procedural workflows and uneven clinical documentation standards collectively cause ENI crude rates to range from 11.2 to 100%. Retrospective observational studies showed a higher pooled effect than prospective designs, likely due to more complete capture of interruption events (24, 33). Developed countries reported higher ENI prevalence than developing countries, possibly related to more frequent procedures and stricter monitoring (12, 21). Gastric feeding alone was associated with higher ENI risk than combined gastric/intestinal feeding, mainly due to frequent GRV monitoring and intolerance (22, 28). In addition, detected publication bias likely inflates the pooled proportion, overstating the overall burden of ENI worldwide. To further quantify the bias induced by divergent ENI duration definitions, we performed stratified leave-one-out sensitivity analyses restricted to clinically meaningful sustained ENI with explicit time thresholds. This stratified sensitivity evidence directly confirms that the inflated global pooled prevalence of 79.2% is largely driven by studies that count all trivial, transient short feeding pauses without standardized minimum duration criteria, rather than prolonged, clinically impactful interruptions that carry substantial risks of cumulative caloric deficits. Instead of relying solely on the single global estimate, clinicians should select subgroup data matching their local ICU context: ENI prevalence is generally higher in high-income countries and units using exclusive gastric feeding, while lower rates are observed in international multicenter cohorts and mixed gastroduodenal feeding protocols. Standardized ENI operational definitions and large prospective multicenter surveillance are required to generate more generalizable incidence data in future research.

The leading causes of ENI were airway management procedures (median 27.1%), radiological/imaging procedures (15.0%), patient-related gastrointestinal factors (16.2%), and surgical procedures with preoperative preparation (10.4%). Airway-related procedures were identified as the most frequent cause of ENI. Although such procedure-related interruptions may be clinically unavoidable, their duration and frequency could still be minimized through standardized protocols, optimized peri-procedural fasting strategies, and prompt resumption of enteral feeding after procedures (6, 44). This also indicates the importance of optimizing peri-procedural feeding strategies, such as minimizing fasting duration and promoting early resumption of feeding. Gastrointestinal intolerance was reported across 14 studies, indicating the presence of modifiable factors that may be addressed through improved feeding protocols and pharmacological interventions. Notably, approximately 13.3% of interruptions were undocumented or unexplained, reflecting inconsistencies in clinical documentation and suggesting the presence of potentially avoidable interruptions. This underscores the need for standardized recording practices and quality improvement initiatives.

Our meta-analysis showed that ENI was significantly associated with lower energy adequacy in critically ill patients, supporting the view that ENI directly compromise nutritional delivery and may contribute to cumulative energy deficits. Previous studies (6, 51) have suggested that inadequate or delayed enteral nutrition may be associated with worse clinical outcomes. However, evidence regarding the timing and intensity of early nutrition should be interpreted according to the phase of critical illness. In a large multicentre prospective cohort study (52), early nutrition support within 48 h of ICU admission was independently associated with increased 28-day mortality, longer ICU lengths of stay, and higher crude mortality, even after multivariable and propensity score adjustments. This finding does not necessarily contradict the present study’s overall thesis. Rather, it suggests that in the very early acute phase, particularly among patients with greater baseline illness severity, hemodynamic instability, shock, or evolving organ dysfunction, ultra-early or aggressive nutritional support may reflect disease severity and may not confer immediate benefit. In this phase, ENI may sometimes be clinically necessary for safety reasons. By contrast, after initial stabilization and during the post-acute or recovery phase, repeated and avoidable ENI may become an important contributor to underfeeding. Insufficient energy delivery during this period can lead to cumulative caloric and protein deficits, which have been consistently associated with adverse outcomes in critically ill patients. The goal is not to promote indiscriminate uninterrupted feeding during all phases of critical illness, but to minimize preventable interruptions and optimize nutritional delivery when EN is clinically appropriate and safe. These considerations provide a more nuanced interpretation of the relationship among early nutrition, ENI, energy adequacy, and prognosis.

For short-term mortality, the pooled RR was 1.86, suggesting a potential increase in risk. However, the 95% CI was extremely wide (0.67–5.11), indicating high imprecision and low statistical power. Similarly, the pooled mean difference for LOS crossed the null value (MD = −0.41, 95% CI −2.05 to 1.23), with overlapping intervals for both shortened and prolonged ICU admission, also reflecting limited ability to draw firm conclusions. Neither result constitutes evidence of no true causal association. These findings should be interpreted with extreme caution for three key reasons. First, only a small number of studies reported data on ICU LOS and short-term mortality separately, severely limiting overall statistical power and the precision of pooled effect estimates. Second, substantial heterogeneity existed for both outcomes, especially mortality (*I*^2^ = 88%), reflecting meaningful clinical and methodological discrepancies across trials that could mask underlying true associations. Third, included work varied widely in enrolled patient cohorts, baseline illness severity, ICU setting, EN operational definitions, and confounder adjustment strategies; short-term mortality endpoints were also inconsistent (in-ICU, 28-day, 30-day timeframes). Varied follow-up windows introduce additional clinical heterogeneity that may dilute any independent link between ENI and hard endpoints.

This contradictory pattern across individual studies is largely attributable to methodological variation. Research detecting harmful outcomes tended to enroll patients with recurrent, long-duration ENI and severe cumulative calorie deficits, or more homogeneous high-risk cohorts where nutritional impairment exerted clearer prognostic effects. In contrast, null findings often stemmed from small sample sizes, overly broad ENI criteria counting trivial transient pauses, or highly mixed ICU populations where multiple competing risk factors diluted the independent impact of ENI. ICU mortality and LOS are multifactorial outcomes driven by numerous non-nutritional variables; rigorous standardization of ENI exposure, confounder adjustment and clinical endpoints is required to isolate the specific prognostic role of ENI. Taken together, our pooled results reflect substantial evidentiary uncertainty rather than proof that ENI has no clinical impact. Large prospective cohorts and RCTs with unified ENI and outcome definitions are necessary to clarify whether minimizing avoidable ENI improves patient-centered clinical outcomes.

Nutritional and biochemical indicators included in this systematic review mainly comprised serum albumin, serum prealbumin, serum total protein, serum hemoglobin, serum transferrin, and serum cholesterol as nutrition-related biomarkers as well as serum alanine transaminase (ALT), serum aspartate aminotransferase (AST), serum total bilirubin, and serum amylase as organ function-related biomarkers. Significant heterogeneity existed in the definition, measurement timing, cutoff values, and reporting methods of the above indicators across studies, with a lack of standardized measurement and reporting criteria. Most studies proposed interventions to cut ENI and optimize nutrition delivery. For higher clinical practicability, future integrated intervention packages should adopt targeted, actionable measures against common avoidable interruptions. Roll out standardized enteral nutrition protocols with clear rules to start or pause feeds and nurse-led feeding workflows. Apply volume-based feeding to make up calorie gaps from repeated feeding breaks and hit daily nutrition targets steadily. Shorten peri-procedural fasting by setting procedure-specific fasting limits and resuming feeds quickly once patients are stable post-procedure. Abolish unnecessary routine GRV checks. Manage GI complications with rational prokinetics, post-pyloric feeding and secure tube care to stop unplanned interruptions. Engage ICU dietitians and multidisciplinary nutrition teams to monitor real-time feeding status, spot preventable pauses and adjust feeding plans timely. These layered, cause-oriented tactics can effectively lower unnecessary ENI and raise feeding sufficiency for critically ill patients.

This study comprehensively searched seven databases, including both English and Chinese literature, and used logit-transformed single-arm meta-analysis to ensure robustness. Subgroup and sensitivity analyses were rigorously performed, and causes and interventions were systematically summarized. However, several limitations should be acknowledged. First, extreme and largely unexplained heterogeneity (*I*^2^ = 99%) was detected across included studies, which was only partially explained by study design and feeding route. Such extensive heterogeneity reflects fundamental differences in case mix, definitions, and clinical practices across studies and cannot be resolved by quantitative pooling, significantly limiting the robustness and clinical utility of the pooled prevalence estimates. Extreme between-study heterogeneity was present for both prevalence and mortality outcomes and could not be fully explained, which further limits the validity and clinical usefulness of our pooled findings. Second, significant publication bias was observed (Egger’s test *p* < 0.001), which may lead to overestimation of the pooled prevalence of ENI. Third, the definition of ENI varied substantially across studies, which limited direct comparability and contributed to heterogeneity. Fourth, the number of studies reporting clinical outcomes was relatively small, with only 3–4 studies included in each meta-analysis of energy adequacy, ICU LOS, and short-term mortality. Fifth, high heterogeneity was observed in the mortality analysis (*I*^2^ = 88%), and most included studies were observational, which limits causal inference between ENI and clinical outcomes. In addition, biochemical marker data were limited, inconsistently reported, and lacked standardized thresholds. Sixth, for short-term mortality, the pooled effect size suggested a potential increase in risk (RR = 1.86), but the wide confidence interval indicated high imprecision and low statistical power. Therefore, the finding reflects uncertainty rather than evidence of no association. Furthermore, our analysis was primarily limited to energy delivery, reflecting the reporting patterns in the included studies. We acknowledge a notable limitation regarding the lack of commentary on protein adequacy. Given recent advancements in critical care nutrition, protein provision is increasingly recognized as being at least as critical as energy delivery for preserving muscle mass and modulating clinical outcomes. Future research should prioritize the rigorous reporting of both energy and protein intake to provide a more comprehensive assessment of nutritional support in the ICU.

These findings have important implications for clinical practice and future research. In clinical practice, targeted interventions should be implemented to reduce the occurrence of ENI, including the establishment of standardized management protocols, reasonable shortening of peri-procedural fasting time, standardized monitoring of gastric residual volume, timely identification and intervention of feeding intolerance, and strengthening the standardized maintenance of feeding tubes. From a research perspective, future studies should aim to improve methodological consistency and evidence quality. The use of standardized definitions of ENI (e.g., ≥10 min) is essential to enhance comparability across studies. Future studies should adopt uniform outcome measures for energy adequacy, ICU LOS, and short-term mortality to reduce heterogeneity. Large-scale prospective cohort studies are needed to better characterize interruption patterns and associated outcomes, while RCTs should evaluate whether reducing ENI can improve nutritional adequacy and clinical outcomes. Furthermore, the exploration of objective biomarkers may help guide individualized feeding strategies, and future research should also focus on developing and evaluating structured quality improvement strategies to reduce avoidable interruptions.

## Conclusion

ENI incidence varied widely across critical care investigations, with a median crude prevalence of 68.5% (IQR: 57.1–96.6%). The global pooled prevalence of 79.2% remains highly uncertain due to extreme heterogeneity and publication bias. Key causes included airway management, radiological procedures, gastrointestinal intolerance, and surgical preparation. ENI was associated with reduced energy adequacy. However, the association between ENI and ICU length of stay as well as short-term mortality remains highly uncertain due to limited outcome data, substantial heterogeneity and wide confidence intervals. Given the limited number of studies and substantial heterogeneity, the impact of ENI on these hard clinical outcomes remains uncertain. Standardized ENI definitions, optimized peri-procedural fasting, improved gastrointestinal management, and multidisciplinary nutrition teams may reduce unnecessary interruptions. Future high-quality studies with unified designs and outcome measures are warranted to optimize EN delivery in critical care.

## Data Availability

The original contributions presented in the study are included in the article/Supplementary material, further inquiries can be directed to the corresponding authors.

## References

[1] Al-Dorzi HM Arabi YM. Nutrition support for critically ill patients. JPEN J Parenter Enteral Nutr. (2021) 45:47–59. doi: 10.1002/jpen.2228, 34897737

[2] Singer P Blaser AR Berger MM Calder PC Casaer M Hiesmayr M . ESPEN practical and partially revised guideline: clinical nutrition in the intensive care unit. Clin Nutr. (2023) 42:1671–89. doi: 10.1016/j.clnu.2023.07.011, 37517372

[3] Chinese Society of Parenteral and Enteral Nutrition (CSPEN). Guideline for clinical application of parenteral and enteral nutrition in adults patients in China (2023 edition). Zhonghua Yi Xue Za Zhi. (2023) 103:946–74. doi: 10.3760/cma.j.cn112137-20221116-02407 36990711

[4] Zhou H-J Zuo D-J Zhang D He X-H Guo S-B. Nutritional status and prognostic factors for mortality in patients admitted to emergency department observation units: a national multi-center study in China. World J Emerg Med. (2023) 14:17–24. doi: 10.5847/wjem.j.1920-8642.2023.005, 36713332 PMC9842475

[5] Tatucu-Babet OA Ridley EJ. How much underfeeding can the critically ill adult patient tolerate? J Intensive Med. (2022) 2:69–77. doi: 10.1016/j.jointm.2022.01.002, 36789187 PMC9923975

[6] Salciute-Simene E Stasiunaitis R Ambrasas E Tutkus J Milkevicius I Sostakaite G . Impact of enteral nutrition interruptions on underfeeding in intensive care unit. Clin Nutr. (2021) 40:1310–7. doi: 10.1016/j.clnu.2020.08.014, 32896448

[7] Page MJ McKenzie JE Bossuyt PM Boutron I Hoffmann TC Mulrow CD . The PRISMA 2020 statement: an updated guideline for reporting systematic reviews. BMJ. (2021) 372:n71. doi: 10.1136/bmj.n71, 33782057 PMC8005924

[8] Wells GA Shea B O’Connell D Peterson J Welch V Losos M . *The Newcastle–Ottawa scale (NOS) for assessing the quality of non-randomised studies in meta-analyses*. Ottawa: Ottawa Hospital Research Institute. (2014). Available online at: https://ohri.ca/en/who-we-are/core-facilities-and-platforms/ottawa-methods-centre/newcastle-ottawa-scale

[9] Ma L-L Wang Y-Y Yang Z-H Huang D Weng H Zeng X-T. Methodological quality (risk of bias) assessment tools for primary and secondary medical studies: what are they and which is better? Mil Med Res. (2020) 7:7. doi: 10.1186/s40779-020-00238-8, 32111253 PMC7049186

[10] Schünemann HJ Oxman AD Brozek J Glasziou P Jaeschke R Vist GE . Grading quality of evidence and strength of recommendations for diagnostic tests and strategies. BMJ. (2008) 336:1106–10. doi: 10.1136/bmj.39500.677199.AE, 18483053 PMC2386626

[11] Han Z Ye XH Zhang R Zuo JT Xu Y Wu CL . Analysis of influencing factors of enteral nutrition interruption and its impact on prognosis in critically ill general surgery patients. Chin J Pract Nurs. (2022) 38:2203–8. doi: 10.3760/cma.j.cn211501-20220228-01402

[12] Zuo JT Li JQ Xu Y Cai LN Wu CL Zheng HY . Current status of enteral nutrition interruption in critically ill patients and its impact on calorie target achievement. Parenter Enteral Nutr. (2023) 30:166–70. doi: 10.16151/j.1007-810x.2023.03.008

[13] Zheng L Sang L Li YM Yan F Liu XQ. Analysis of risk factors for inadequate enteral feeding in critically ill patients. Chin J Resp Crit Care Med. (2014) 13:394–7. doi: 10.7507/1671-6205.2014096

[14] Gong XM. A survey study on the current status of early enteral nutrition feeding interruption in critically ill patients. Chin J Mod Nurs. (2019) 25:1646. doi: 10.3760/cma.j.issn.1674-2907.2019.13.013

[15] Fang YY. Causes and countermeasures of early enteral nutrition interruption in critically ill patients with stroke. J Emerg Trad Chin Med. (2012) 21:849–50. doi: 10.3969/j.issn.1004-745X.2012.05.115

[16] Wang L Li Y. Risk of enteral nutrition interruption and construction of a prediction model in mechanically ventilated patients with severe stroke. J Med Theory Pract. (2025) 38:323–5. doi: 10.19381/j.issn.1001-7585.2025.02.049

[17] Li N. *Current status and influencing factors of feeding intolerance in patients with severe neurological diseases* (master’s thesis). Lanzhou University (2021). Available online at: https://med.wanfangdata.com.cn/Paper/Detail?id=DegreePaper_D02336905&dbid=WF_XW (accessed March 27, 2026)

[18] Ribeiro LMK Oliveira Filho RS Caruso L Lima PA Damasceno NRT Soriano FG. Adequacy of energy and protein balance of enteral nutrition in intensive care: what are the limiting factors? Rev Bras Ter Intensiva. (2014) 26:155–62. doi: 10.5935/0103-507X.20140023, 25028950 PMC4103942

[19] Kim H Shin JA Shin JY Cho OM. Adequacy of nutritional support and reasons for underfeeding in neurosurgical intensive care unit patients. Asian Nurs Res. (2010) 4:102–10. doi: 10.1016/S1976-1317(10)60010-2, 25030950

[20] Peev MP Yeh DD Quraishi SA Osler P Chang Y Gillis E . Causes and consequences of interrupted enteral nutrition: a prospective observational study in critically ill surgical patients. J Parenter Enter Nutr. (2015) 39:21–7. doi: 10.1177/0148607114526887, 24714361 PMC4402286

[21] Zheng H Cai L Wang P Zheng L Lin J Sun T . Causes and impacts of interrupted enteral nutrition in critically ill patients: a secondary analysis of a cluster‐randomized controlled trial. Nurs Crit Care. (2025) 30:e70006. doi: 10.1111/nicc.70006 40069998

[22] Pinilla JC Samphire J Arnold C Liu L Thiessen B. Comparison of gastrointestinal tolerance to two enteral feeding protocols in critically III patients: a prospective, randomized controlled trial. J Parenter Enter Nutr. (2001) 25:81–6. doi: 10.1177/014860710102500281, 11284474

[23] Daphnee DK Bhuvaneshwari S. Critical care 24 × 7: but, why is critical nutrition interrupted? Indian J Crit Care Med. (2014) 18:144–8. doi: 10.4103/0972-5229.128704, 24701064 PMC3963197

[24] Binnekade J Tepaske R Bruynzeel P Mathus-Vliegen E De Haan R. Daily enteral feeding practice on the ICU: attainment of goals and interfering factors. Crit Care. (2005) 9:R218–25. doi: 10.1186/cc3504, 15987393 PMC1175883

[25] Davies C Johansson L Brett SJ Cole E. Enteral nutrition delivery in patients admitted to a critical care unit following major trauma: who’s at risk and what’s the impact? J Intensive Care Soc. (2025) 26:435–45. doi: 10.1177/17511437251349679, 40600217 PMC12206743

[26] Onuk S Ozer NT Savas N Sipahioglu H Temel S Ergul SS . Enteral nutrition interruptions in critically ill patients: a prospective study on reasons, frequency and duration of interruptions of nutritional support during ICU stay. Clin Nutr ESPEN. (2022) 52:178–83. doi: 10.1016/j.clnesp.2022.10.019, 36513451

[27] Van Nieuwkoop MM Ramnarain D Pouwels S. Enteral nutrition interruptions in the intensive care unit: a prospective study. Nutrition. (2022) 96:111580. doi: 10.1016/j.nut.2021.111580, 35101813

[28] Kim H Stotts NA Froelicher ES Engler MM Porter C. Enteral nutritional intake in adult korean intensivecare patients. Am J Crit Care. (2013) 22:126–35. doi: 10.4037/ajcc2013629, 23455862 PMC4748854

[29] McClave SA Sexton LK Spain DA Adams JL Owens NA Sullins MB . Enteral tube feeding in the intensive care unit: factors impeding adequate delivery. Crit Care Med. (1999) 27:1252–6. doi: 10.1097/00003246-199907000-00003, 10446815

[30] O’Meara D Mireles-Cabodevila E Frame F Hummell AC Hammel J Dweik RA . Evaluation of delivery of enteral nutrition in critically ill patients receiving mechanical ventilation. Am J Crit Care. (2008) 17:53–61. doi: 10.4037/ajcc2008.17.1.53, 18158390

[31] Yip KF Rai V Wong KK. Evaluation of delivery of enteral nutrition in mechanically ventilated Malaysian ICU patients. BMC Anesthesiol. (2014) 14:127. doi: 10.1186/1471-2253-14-127, 25587238 PMC4292820

[32] Kasti A Theodorakopoulou M Katsas K Synodinou K Nikolaki M Zouridaki A . Factors associated with interruptions of enteral nutrition and the impact on macro- and micronutrient deficits in ICU patients. Nutrients. (2023) 15:917. doi: 10.3390/nu15040917, 36839275 PMC9959226

[33] Morgan LM Dickerson RN Alexander KH Brown RO Minard G. Factors causing interrupted delivery of enteral nutrition in trauma intensive care unit patients. Nut Clin Prac. (2004) 19:511–7. doi: 10.1177/0115426504019005511, 16215147

[34] Shankar B Daphnee DK Ramakrishnan N Venkataraman R. Feasibility, safety, and outcome of very early enteral nutrition in critically ill patients: results of an observational study. J Crit Care. (2015) 30:473–5. doi: 10.1016/j.jcrc.2015.02.009, 25791768

[35] Saran D Brody RA Stankorb SM Parrott SJ Heyland DK. Gastric vs small bowel feeding in critically ill neurologically injured patients: results of a Multicenter observational study. JPEN J Parenter Enteral Nutr. (2015) 39:910–6. doi: 10.1177/0148607114540003, 24947058

[36] Czapran A Headdon W Deane AM Lange K Chapman MJ Heyland DK. International observational study of nutritional support in mechanically ventilated patients following burn injury. Burns. (2015) 41:510–8. doi: 10.1016/j.burns.2014.09.013, 25445003

[37] Coutris N Gawaziuk JP Cristall N Logsetty S. Interrupted nutrition support in patients with burn injuries: a single-Centre observational study. Plast Surg (Oakv). (2019) 27:334–9. doi: 10.1177/2292550319880917, 31763334 PMC6851732

[38] Uozumi M Sanui M Komuro T Iizuka Y Kamio T Koyama H . Interruption of enteral nutrition in the intensive care unit: a single-center survey. J Intensive Care. (2017) 5:52. doi: 10.1186/s40560-017-0245-9, 28794882 PMC5545034

[39] Habib M Murtaza HG Kharadi N Mehreen T Ilyas A Khan AH . Interruptions to enteral nutrition in critically ill patients in the intensive care unit. Cureus. (2022) 14:e22821. doi: 10.7759/cureus.22821, 35382187 PMC8976564

[40] Lakshmi R Vaidyanathan R. Nutritional adequacy in intensive care patients—a pilot study. Int J Nurs Educ. (2012) 4:60–2.

[41] Arunkumar A Kalaiselvan M Renuka M Sivakumar R. Nutritional adequacy in mechanically ventilated patient: are we doing enough? Indian J Crit Care Med. (2021) 25:166–71. doi: 10.5005/jp-journals-10071-23717, 33707894 PMC7922459

[42] O’Leary-Kelley CM Puntillo KA Barr J Stotts N Douglas MK. Nutritional adequacy in patients receiving mechanical ventilation who are fed enterally. Am J Crit Care. (2005) 14:222–31. doi: 10.4037/ajcc2005.14.3.222, 15840896

[43] Davies AR Morrison SS Ridley EJ Bailey M Banks MD Cooper DJ . Nutritional therapy in patients with acute pancreatitis requiring critical care unit management: a prospective observational study in Australia and New Zealand*. Crit Care Med. (2011) 39:462–8. doi: 10.1097/CCM.0b013e318205df6d, 21221003

[44] Segaran E Barker I Hartle A. Optimising enteral nutrition in critically ill patients by reducing fasting times. J Intensive Care Soc. (2016) 17:38–43. doi: 10.1177/1751143715599410, 28979456 PMC5606376

[45] Elpern EH Stutz L Peterson S Gurka DP Skipper A. Outcomes associated with enteral tube feedings in a medical intensive care unit. Am J Crit Care. (2004) 13:221–7. doi: 10.4037/ajcc2004.13.3.221, 15149056

[46] Passier RHA Davies AR Ridley E McClure J Murphy D Scheinkestel CD. Periprocedural cessation of nutrition in the intensive care unit: opportunities for improvement. Intensive Care Med. (2013) 39:1221–6. doi: 10.1007/s00134-013-2934-8, 23636828

[47] Lee Z-Y Ibrahim NA Mohd-Yusof B-N. Prevalence and duration of reasons for enteral nutrition feeding interruption in a tertiary intensive care unit. Nutrition. (2018) 53:26–33. doi: 10.1016/j.nut.2017.11.014, 29627715

[48] Williams TA Leslie GD Leen T Mills L Dobb GJ. Reducing interruptions to continuous enteral nutrition in the intensive care unit: a comparative study. J Clin Nurs. (2013) 22:2838–48. doi: 10.1111/jocn.12068, 23311643

[49] Ritter CG Medeiros IMS Pádua CSD Gimenes FRE Prado PRD. Risk factors for protein-caloric inadequacy in patients in an intensive care unit. Rev Bras Ter Intensiva. (2019) 31:504–10. doi: 10.5935/0103-507X.20190067, 31967225 PMC7008981

[50] Rice TW Swope T Bozeman S Wheeler AP. Variation in enteral nutrition delivery in mechanically ventilated patients. Nutrition. (2005) 21:786–92. doi: 10.1016/j.nut.2004.11.014, 15975485

[51] Stewart ML. Interruptions in enteral nutrition delivery in critically ill patients and recommendations for clinical practice. Crit Care Nurse. (2014) 34:14–22; (quiz 22). doi: 10.4037/ccn2014243, 25086090

[52] Pardo E Lescot T Preiser JC Massanet P Pons A Jaber S . Association between early nutrition support and 28-day mortality in critically ill patients: the FRANS prospective nutrition cohort study. Crit Care. (2023) 27:7. doi: 10.1186/s13054-022-04298-1, 36611211 PMC9826592

