# Supplementary material for: Enteral nutrition interruption in critically ill patients: a systematic review and meta-analysis of prevalence, causes, and clinical outcomes
Source: Front Nutr. 2026 Sep 24;13:1886962. doi: 10.3389/fnut.2026.1886962 (PMC13645539; doi:10.3389/fnut.2026.1886962)
Supplement: Supplementary file 1 [file Data_Sheet_1.docx]

**Supplementary Materials**

Catalogue

[Search strategy	1](#_Toc4836)

[Table S1. Search strategy of PubMed	1](#_Toc9883)

[Table S2. Search strategy of Cochrane Library	2](#_Toc22221)

[Table S3. Search strategy of Embase	2](#_Toc2212)

[Table S4. Search strategy of Web of Science Core Collection	3](#_Toc24200)

[Table S5. Search strategy of CINAHL Plus with Full Text	3](#_Toc17040)

[Table S6. Search strategy of CNKI	4](#_Toc11661)

[Table S7. Search strategy of WanFang Data	4](#_Toc3786)

[Quality evaluation of the literature	5](#_Toc3615)

[Table S8. Quality assessment of cohort and observational studies (Newcastle-Ottawa Scale, NOS)	5](#_Toc18924)

[Table S9. Quality assessment of cross-sectional studies (AHRQ checklist)	5](#_Toc16466)

[Table S10. Quality assessment of randomized controlled trials (RCTs) (Cochrane ROB 2.0)	5](#_Toc6703)

[Table S11 Categorized summary of ENI duration definition subgroups	7](#_Toc26146)

[Table S12 Summary of Sensitivity Analyses for ENI ≥10 min Subgroup	7](#_Toc919)

[Table S13 Summary of Sensitivity Analyses for ENI ≥15 min Subgroup	7](#_Toc15737)

[Table S14 Summary of Sensitivity Analyses for ENI ≥30 min Subgroup	8](#_Toc22146)

[Subgroup analysis	10](#_Toc32524)

[Figure S1. Forest plot of subgroup analysis by ENI time threshold definition	10](#_Toc4975)

[Figure S2. Forest plot of subgroup analysis by ICU type	11](#_Toc12663)

[Figure S3. Forest plot of subgroup analysis by feeding route	12](#_Toc30266)

[Figure S4 The Leave-one-study sensitivity analyses	13](#_Toc31245)

[Figure S5.Funnel plot of publication bias assessment	13](#_Toc11311)

[Figure S6.The Egger’s regression plot	14](#_Toc296)

[Figure S7. Forest plot of sensitivity analysis excluding the study with the most extreme effect size	14](#_Toc5010)

**Search strategy**

Table S1. Search strategy of PubMed

| **Category** | **No.** | **Search strategy** | **Results** |
| --- | --- | --- | --- |
| Population | #1 | "Intensive Care Units"[MeSH Terms] OR "ICU patients"[Title/Abstract] OR "critically ill patients"[Title/Abstract] OR "Critical patients"[Title/Abstract] OR "critical care"[Title/Abstract] OR "Critical ill"[Title/Abstract] OR "intensive care"[Title/Abstract] | 320,727 |
| Intervention | #2 | "Enteral Nutrition"[MeSH Terms] OR "Enteral feeding"[Title/Abstract] OR "nutrition support"[Title/Abstract] OR "nutrition delivery"[Title/Abstract] OR "nutritional intake"[Title/Abstract] OR "Nutrition intervention"[Title/Abstract] OR "tube feeding*"[Title/Abstract] | 37,185 |
| Outcome | #3 | "Treatment Interruption"[MeSH Terms] OR "feeding interruption"[Title/Abstract] OR "nutrition interruption"[Title/Abstract] OR "enteral nutrition disruption"[Title/Abstract] OR "nutrition* interruption"[Title/Abstract] OR "enteral nutrition* interruption"[Title/Abstract] OR "nutrition support* interruption"[Title/Abstract] OR "interrupt*"[Title/Abstract] OR "interruption"[Title/Abstract] OR "disrupt*"[Title/Abstract] OR "disruption"[Title/Abstract] OR "influenc*"[Title/Abstract] | 2,564,248 |
| P+I+O | #4 | #1 AND #2 AND #3 | 583 |

Table S2. Search strategy of Cochrane Library

| **Category** | **No.** | **Search strategy** | **Results** |
| --- | --- | --- | --- |
| Population | #1 | "Intensive Care Units"[MeSH descriptor, explode all trees] OR "ICU patients"[ti,ab,kw] OR "critically ill patients"[ti,ab,kw] OR "Critical patients"[ti,ab,kw] OR "critical care"[ti,ab,kw] OR "Critical ill"[ti,ab,kw] OR "intensive care"[ti,ab,kw] | 73,601 |
| Intervention | #2 | "Enteral Nutrition"[MeSH descriptor, explode all trees] OR "Enteral feeding"[ti,ab,kw] OR "nutrition support"[ti,ab,kw] OR "nutrition delivery"[ti,ab,kw] OR "nutritional intake"[ti,ab,kw] OR "Nutrition intervention"[ti,ab,kw] OR "tube feeding*"[ti,ab,kw] | 34,009 |
| Outcome | #3 | "Treatment Interruption"[MeSH descriptor, explode all trees] OR "feeding interruption"[ti,ab,kw] OR "nutrition interruption"[ti,ab,kw] OR "enteral nutrition disruption"[ti,ab,kw] OR "nutrition* interruption"[ti,ab,kw] OR "enteral nutrition* interruption"[ti,ab,kw] OR "nutrition support* interruption"[ti,ab,kw] OR "interrupt*"[ti,ab,kw] OR "interruption"[ti,ab,kw] OR "disrupt*"[ti,ab,kw] OR "disruption"[ti,ab,kw] OR "influenc*"[ti,ab,kw] | 136,833 |
| P+I+O | #4 | #1 AND #2 AND #3 | 432 |

Table S3. Search strategy of Embase

| **Category** | **No.** | **Search strategy** | **Results** |
| --- | --- | --- | --- |
| Population | #1 | "intensive care unit"/exp OR "intensive care unit" OR "intensive care"/exp OR "intensive care" OR "icu patients":ab,ti OR "critically ill patients":ab,ti OR "critical patients":ab,ti OR "critical care":ab,ti OR "critical ill":ab,ti OR "intensive care":ab,ti | 1,551,359 |
| Intervention | #2 | "enteric feeding"/exp OR "enteric feeding" OR "enteral feeding":ab,ti OR "nutrition support":ab,ti OR "nutrition delivery":ab,ti OR "nutritional intake":ab,ti OR "nutrition intervention":ab,ti OR "tube feeding*":ab,ti | 65,974 |
| Outcome | #3 | "treatment interruption"/exp OR "treatment interruption" OR "feeding interruption":ab,ti OR "nutrition interruption":ab,ti OR "enteral nutrition disruption":ab,ti OR "nutrition* interruption":ab,ti OR "enteral nutrition* interruption":ab,ti OR "nutrition support* interruption":ab,ti OR "interrupt*":ab,ti OR "interruption":ab,ti OR "disrupt*":ab,ti OR "disruption":ab,ti OR "influenc*":ab,ti | 3,258,958 |
| P+I+O | #4 | #1 AND #2 AND #3 | 1,010 |

Table S4. Search strategy of Web of Science Core Collection

| **Category** | **No.** | **Search strategy** | **Results** |
| --- | --- | --- | --- |
| Population | #1 | TS=("Intensive Care Units" OR "ICU patients" OR "critically ill patients" OR "Critical patients" OR "critical care" OR "Critical ill" OR "intensive care") | 569,735 |
| Intervention | #2 | TS=("Enteral Nutrition" OR "Enteral feeding" OR "nutrition support" OR "nutrition delivery" OR "nutritional intake" OR "Nutrition intervention" OR "tube feeding*") | 144,909 |
| Outcome | #3 | TS=("Treatment Interruption" OR "feeding interruption" OR "nutrition interruption" OR "enteral nutrition disruption" OR "nutrition* interruption" OR "enteral nutrition* interruption" OR "nutrition support* interruption" OR "interrupt*" OR "interruption" OR "disrupt*" OR "disruption" OR "influenc*") | 4,527,306 |
| P+I+O | #4 | #1 AND #2 AND #3 | 1,886 |

Table S5. Search strategy of CINAHL Plus with Full Text

| **Category** | **No.** | **Search strategy** | **Results** |
| --- | --- | --- | --- |
| Population | #1 | SU="Intensive Care Units" OR XB=("ICU patients" OR "critically ill patients" OR "Critical patients" OR "critical care" OR "Critical ill" OR "intensive care") | 170,221 |
| Intervention | #2 | SU="Enteral Nutrition" OR XB=("Enteral feeding" OR "nutrition support" OR "nutrition delivery" OR "nutritional intake" OR "Nutrition intervention" OR "tube feeding*") | 428,368 |
| Outcome | #3 | SU="Treatment Interruption" OR XB=("feeding interruption" OR "nutrition interruption" OR "enteral nutrition disruption" OR "nutrition* interruption" OR "enteral nutrition* interruption" OR "nutrition support* interruption" OR "interrupt*" OR "interruption" OR "disrupt*" OR "disruption" OR "influenc*") | 10669 |
| P+I+O | #4 | #1 AND #2 AND #3 | 1,052 |

Table S6. Search strategy of CNKI

| **Category** | **No.** | **Search strategy** | **Results** |
| --- | --- | --- | --- |
| Population | #1 | 篇关摘: ICU + 危重症患者 + 重症患者 + 重症监护 (精确) | - |
| Intervention | #2 | 篇关摘：肠内营养 + 营养支持 + 营养输送 + 营养摄入 + 营养干预 + 管饲 (精确) | - |
| Outcome | #3 | 篇关摘：营养中断 + 喂养中断 + 治疗中断 + 喂养中止 + 中断 + 扰乱 (精确) | - |
| P+I+O | #4 | #1 AND #2 AND #3 | 97 |

Table S7. Search strategy of WanFang Data

| **Category** | **No.** | **Search strategy** | **Results** |
| --- | --- | --- | --- |
| Population | #1 | 题名或关键词: ICU OR 危重症患者 OR 重症患者 OR 重症监护 | - |
| Intervention | #2 | 题名或关键词：肠内营养 OR 营养支持 OR 营养输送 OR 营养摄入 OR 营养干预 OR 管饲 | - |
| Outcome | #3 | 题名或关键词：营养中断 OR 喂养中断 OR 治疗中断 OR 喂养中止 OR 中断 OR 扰乱 | - |
| P+I+O | #4 | #1 AND #2 AND #3 | 53 |

**Quality evaluation of the literature**

Table S8. Quality assessment of cohort and observational studies (Newcastle-Ottawa Scale, NOS)

| **Study** | **Selection (4)** | **Comparability (2)** | **Outcome (3)** | **Total score** |
| --- | --- | --- | --- | --- |
| Arezina 2023 | ★★★★ | ★ | ★★★ | 8 |
| Arunkumar 2021 | ★★★★ | ★ | ★★★ | 8 |
| Binnekade 2005 | ★★★★ | ★ | ★★★ | 8 |
| Davies 2025 | ★★★★ | ★ | ★★★ | 8 |
| Celso 2019 | ★★★ | ★ | ★★★ | 7 |
| Coutris 2019 | ★★★ | ★ | ★★★ | 7 |
| Czapran 2015 | ★★★★ | ★ | ★★★ | 8 |
| Davies 2011 | ★★★★ | ★ | ★★★ | 8 |
| Ella 2016 | ★★★★ | ★ | ★★★ | 8 |
| Elpern 2004 | ★★★★ | ★ | ★★★ | 8 |
| Erika 2021 | ★★★★ | ★ | ★★★ | 8 |
| Habib 2022 | ★★★★ | ★ | ★★★ | 8 |
| Han 2022 | ★★★ | ★ | ★★★ | 7 |
| Kim 2010 | ★★★★ | ★ | ★★★ | 8 |
| Kim 2013 | ★★★★ | ★ | ★★★ | 8 |
| Lakshmi 2012 | ★★★★ | ★ | ★★★ | 8 |
| Leary 2005 | ★★★★ | ★ | ★★★ | 8 |
| Lia Mara 2014 | ★★★★ | ★ | ★★★ | 8 |
| McClave 1999 | ★★★★ | ★ | ★★★ | 8 |
| O’Meara 2008 | ★★★★ | ★ | ★★★ | 8 |
| Midor 2017 | ★★★ | ★ | ★★★ | 7 |
| Miroslav 2015 | ★★★★ | ★ | ★★★ | 8 |
| Monica 2022 | ★★★★ | ★ | ★★★ | 8 |
| Morgan 2004 | ★★★ | ★ | ★★★ | 7 |
| Nagarajan 2014 | ★★★★ | ★ | ★★★ | 8 |
| Roeland 2013 | ★★★ | ★ | ★★★ | 7 |
| Saran 2015 | ★★★★ | ★ | ★★★ | 8 |
| Sevda Onuk 2022 | ★★★★ | ★ | ★★★ | 8 |
| Shankar 2015 | ★★★★ | ★ | ★★★ | 8 |
| Teresa 2013 | ★★★★ | ★ | ★★★ | 8 |
| Todd 2005 | ★★★★ | ★ | ★★★ | 8 |
| Wang Li 2025 | ★★★★ | ★ | ★★★ | 8 |
| Zheng Lei 2014 | ★★★★ | ★ | ★★★ | 8 |
| Zheng-Yii Lee 2018 | ★★★★ | ★ | ★★★ | 8 |
| Zuo Juntao 2023 | ★★★ | ★ | ★★★ | 7 |

Table S9. Quality assessment of cross-sectional studies (AHRQ checklist)

| **Study** | **Q1** | **Q2** | **Q3** | **Q4** | **Q5** | **Q6** | **Q7** | **Q8** | **Q9** | **Q10** | **Q11** | **Score** |
| --- | --- | --- | --- | --- | --- | --- | --- | --- | --- | --- | --- | --- |
| Fang 2012 | 1 | 1 | 1 | 1 | 1 | 0 | 1 | 1 | 1 | 0 | 1 | 9 |
| Gong 2019 | 1 | 1 | 1 | 1 | 1 | 1 | 1 | 1 | 1 | 0 | 1 | 10 |
| Li Na 2021 | 1 | 1 | 1 | 1 | 1 | 0 | 1 | 1 | 1 | 0 | 1 | 9 |
| Yip 2014 | 1 | 1 | 1 | 1 | 1 | 0 | 1 | 1 | 1 | 0 | 1 | 9 |

Table S10. Quality assessment of randomized controlled trials (RCTs) (Cochrane ROB 2.0)

| **Study** | **Randomization** | **Allocation concealment** | **Blinding participants** | **Blinding outcome assessment** | **Incomplete data** | **Selective reporting** | **Overall risk** |
| --- | --- | --- | --- | --- | --- | --- | --- |
| Jaime 2001 | Low | Low | High | Low | Low | Low | Some concerns |
| Zheng 2025 | Low | Low | High | Low | Low | Low | Some concerns |

Table S11 Categorized summary of ENI duration definition subgroups

| **ENI duration definition subgroup** | **Number of included studies (total n=41)** | **Raw ENI prevalence range of individual studies** | **Total sample size** |
| --- | --- | --- | --- |
| No standardized duration threshold reported | 32 (78.1%) | 11.2%–100% | 6307 |
| Interruption ≥10 minutes | 3 (7.3%) | 64.8%–100% | 331 |
| Interruption ≥15 minutes | 3 (7.3%) | 68.5%–98.3% | 316 |
| Interruption ≥30 minutes (including ≥1 hour criterion) | 3 (7.3%) | 18.6%–79.2% | 1306 |
| Total | 41 (100.0%) | 11.2%–100% | 8260 |

Table S12 Summary of Sensitivity Analyses for ENI ≥10 min Subgroup

| **Analysis Method** | **Effect Model** | **Pooled OR** | **95% Confidence Interval** | **P value** | **Heterogeneity I2** | **Result Judgment** |
| --- | --- | --- | --- | --- | --- | --- |
| All 3 studies included (Baseline) | Random-effects Model | 2.93 | [1.23, 6.99] | 0.02 | 82% | Significant positive effect, CI does not cross null line 1 |
| All 3 studies included (Baseline) | Fixed-effects Model | 2.08 | [1.57, 2.77] | ＜0.00001 | 82% | Significant positive effect, highly overlapping with RE CI |
| Excluded Han Zhen et al. 2022 | Random-effects Model | 17.95 | [0.19, 1729.48] | 0.22 | 91% | CI crosses null line 1, non-significant |
| Excluded Han Zhen et al. 2022 | Fixed-effects Model | 2.36 | [1.57, 3.53] | ＜0.00001 | 91% | Still significant positive, core conclusion unchanged |
| Excluded Miroslav et al. 2015 | Random-effects Model | 16.81 | [0.15, 1867.55] | 0.24 | 91% | CI crosses null line 1, non-significant |
| Excluded Miroslav et al. 2015 | Fixed-effects Model | 2.04 | [1.37, 3.03] | 0.0004 | 91% | Still significant positive, core conclusion unchanged |
| Excluded Zuo Juntao et al. 2023 (Outlier) | Random-effects Model | 1.98 | [1.49, 2.64] | ＜0.00001 | 0% | Significant positive, within-group heterogeneity fully eliminated |
| Excluded Zuo Juntao et al. 2023 (Outlier) | Fixed-effects Model | 1.98 | [1.49, 2.64] | ＜0.00001 | 0% | Identical results under two models, highly robust |

Table S13 Summary of Sensitivity Analyses for ENI ≥15 min Subgroup

| **Analysis Method** | **Effect Model** | **Pooled OR** | **95% Confidence Interval** | **P value** | **Heterogeneity I2** | **Result Judgment** |
| --- | --- | --- | --- | --- | --- | --- |
| All 3 studies included (Baseline) | Random-effects Model | 20.04 | [1.13, 353.94] | 0.04 | 89% | Significant positive effect, CI does not cross null line 1 |
| All 3 studies included (Baseline) | Fixed-effects Model | 2.42 | [1.85, 3.18] | ＜0.00001 | 89% | Significant positive effect, highly overlapping with RE CI |
| Excluded Gong Xuemei et al. 2019 | Random-effects Model | 9.78 | [0.39, 243.15] | 0.16 | 90% | CI crosses null line 1, non-significant |
| Excluded Gong Xuemei et al. 2019 | Fixed-effects Model | 2.34 | [1.78, 3.07] | ＜0.00001 | 90% | Still significant positive, core conclusion unchanged |
| Excluded Leary et al. 2005 | Random-effects Model | 12.46 | [0.26, 594.82] | 0.20 | 87% | CI crosses null line 1, non-significant |
| Excluded Leary et al. 2005 | Fixed-effects Model | 2.28 | [1.73, 3.00] | ＜0.00001 | 87% | Still significant positive, core conclusion unchanged |
| Excluded Meara et al. 2008 (Outlier) | Random-effects Model | 74.22 | [14.81, 371.80] | ＜0.00001 | 0% | Significant positive, within-group heterogeneity fully eliminated |
| Excluded Meara et al. 2008 (Outlier) | Fixed-effects Model | 74.22 | [14.81, 371.80] | ＜0.00001 | 0% | Identical results under two models, highly robust |

Table S14 Summary of Sensitivity Analyses for ENI ≥30 min Subgroup

| **Analysis Method** | **Effect Model** | **Pooled OR** | **95% Confidence Interval** | **P value** | **Heterogeneity I2** | **Result Judgment** |
| --- | --- | --- | --- | --- | --- | --- |
| All 3 studies included (Baseline) | Random-effects Model | 1.04 | [0.18, 6.05] | 0.97 | 99% | Non-significant, CI crosses null line 1 |
| All 3 studies included (Baseline) | Fixed-effects Model | 0.26 | [0.24, 0.28] | ＜0.00001 | 99% | Significant positive, no overlap with RE CI |
| Excluded Hengyu Zheng et al. 2025 (Outlier) | Random-effects Model | 2.22 | [0.80, 6.20] | 0.13 | 90% | CI still crosses null line 1, non-significant |
| Excluded Hengyu Zheng et al. 2025 (Outlier) | Fixed-effects Model | 1.97 | [1.44, 2.70] | ＜0.00001 | 90% | Significant positive, core conclusion unchanged |
| Excluded Wang Li et al. 2025 | Random-effects Model | 0.92 | [0.06, 14.47] | 0.95 | 99% | CI crosses null line 1, non-significant |
| Excluded Wang Li et al. 2025 | Fixed-effects Model | 0.24 | [0.23, 0.26] | ＜0.00001 | 99% | Significant positive, core conclusion unchanged |
| Excluded Yip et al. 2014 | Random-effects Model | 0.55 | [0.10, 3.08] | 0.49 | 99% | CI crosses null line 1, non-significant |
| Excluded Yip et al. 2014 | Fixed-effects Model | 0.24 | [0.23, 0.26] | ＜0.00001 | 99% | Significant positive, core conclusion unchanged |

**Subgroup analysis**

Figure S1. Forest plot of subgroup analysis by ENI time threshold definition

Note: This figure presents the subgroup analysis stratified by the definition of enteral nutrition interruption (ENI) time threshold, including unreported, ≥10 minutes, ≥15 minutes, and ≥30 minutes. The test for subgroup differences shows no significant difference in effect estimates across subgroups (Chi² = 3.55, df = 3, P = 0.31, I² = 15.4%).

Figure S2. Forest plot of subgroup analysis by ICU type

Note: This figure presents the subgroup analysis stratified by intensive care unit (ICU) type, including general ICU, medical ICU, neurocritical ICU, and surgical ICU. The test for subgroup differences shows no significant difference in effect estimates across ICU types (Chi² = 1.23, df = 3, P = 0.75, I² = 0%).

Figure S3. Forest plot of subgroup analysis by feeding route

Note: This figure presents the subgroup analysis stratified by enteral nutrition administration routes, including continuous infusion, intermittent feeding, progressive continuous infusion, and unreported route. The test for subgroup differences shows no significant difference in effect estimates across subgroups (Chi² = 2.61, df = 3, P = 0.46, I² = 0%).

Figure S4 The Leave-one-study sensitivity analyses

Figure S5.Funnel plot of publication bias assessment

Figure S6.The Egger’s regression plot

Figure S7. Forest plot of sensitivity analysis excluding the study with the most extreme effect size

Note: This figure presents the sensitivity analysis conducted by omitting the study with the most extreme effect size (Carys Davies et al., 2025). After exclusion, heterogeneity was reduced (I² = 34%, P = 0.22), and the pooled risk ratio (RR = 1.00, 95% CI: 0.67–1.49, P = 1.00) confirmed the robustness of the primary finding of no significant association between ENI and the outcome.
